# The tepary bean genome provides insight into evolution and domestication under heat stress

**DOI:** 10.1038/s41467-021-22858-x

**Published:** 2021-05-11

**Authors:** Samira Mafi Moghaddam, Atena Oladzad, Chushin Koh, Larissa Ramsay, John P. Hart, Sujan Mamidi, Genevieve Hoopes, Avinash Sreedasyam, Andrew Wiersma, Dongyan Zhao, Jane Grimwood, John P. Hamilton, Jerry Jenkins, Brieanne Vaillancourt, Joshua C. Wood, Jeremy Schmutz, Sateesh Kagale, Timothy Porch, Kirstin E. Bett, C. Robin Buell, Phillip E. McClean

**Affiliations:** 1 Department of Plant Biology, Michigan State University, East Lansing, MI USA; 2 Plant Resilience Institute, Michigan State University, East Lansing, MI USA; 3 Department of Plant Sciences and Genomics and Bioinformatics Program, North Dakota State University, Fargo, ND USA; 4 Department of Plant Sciences, University of Saskatchewan, Saskatoon, SK Canada; 5 Global Institute for Food Security (GIFS), University of Saskatchewan, Saskatoon, SK Canada; 6 USDA-ARS-Tropical Agriculture Research Station, Mayaguez, PR USA; 7 HudsonAlpha Institute for Biotechnology, Huntsville, AL USA; 8 US Department of Energy Joint Genome Institute, Lawrence Berkeley National Laboratory, Berkeley, CA USA; 9 National Research Council Canada, Saskatoon, SK Canada; 10 Michigan State University AgBioResearch, East Lansing, MI USA

**Keywords:** Agricultural genetics, Genome, Comparative genomics, Plant genetics

## Abstract

Tepary bean (*Phaseolus acutifolis* A. Gray), native to the Sonoran Desert, is highly adapted to heat and drought. It is a sister species of common bean (*Phaseolus vulgaris* L.), the most important legume protein source for direct human consumption, and whose production is threatened by climate change. Here, we report on the tepary genome including exploration of possible mechanisms for resilience to moderate heat stress and a reduced disease resistance gene repertoire, consistent with adaptation to arid and hot environments. Extensive collinearity and shared gene content among these *Phaseolus* species will facilitate engineering climate adaptation in common bean, a key food security crop, and accelerate tepary bean improvement.

## Abstract (web summary)

In contrast to common bean, tepary bean is highly adapted to heat and drought. Here, the authors assemble the genomes of tepary bean landrace and wild accession, discuss the possible mechanism for resilience to heat stress, and reveal a reduced disease resistance gene repertoire.

## Introduction

Heat and drought threaten global food security due to their impact on crop productivity. While genetic adaptations to these stresses can be found in plant species that evolved under extreme climatic conditions, few domesticated crops show economically sustainable food production when exposed to abiotic stress. To increase the productivity of a modern crop for a warmer and drier climate, it is important to discover the variation in either the crop species or close relatives for which genetic variation for fitness under abiotic stress has evolved.

Tepary bean (*Phaseolus acutifolius* A. Gray) recently gained attention in modern crop improvement efforts as a source of genetic traits for biotic and abiotic stress resistance for its sister species, common bean (*P. vulgaris* L.). Common bean, the most consumed grain legume worldwide, is adapted to a wide range of climates, but is sensitive to drought and extreme temperatures, which result in significant yield reduction under those environmental conditions^1–4^. The close phylogenetic relationship between tepary bean and common bean enables tepary bean to serve as a key source of heat and drought tolerance. While hybridization between these two species is difficult, persistent breeding efforts have successfully transferred genes conferring common bacterial blight resistance^5,6^, bruchid seed pest resistance^7^, and drought tolerance^8^ from tepary to common bean.

Considering the rapid rate of climate change documented worldwide, the adaptation of tepary bean to high ambient temperatures and to the dry, arid conditions of its native Sonoran Desert habitat^9^ positions it as an alternative protein source in areas threatened with a hotter and drier future. Tepary bean is currently produced on a limited scale, primarily by subsistence farmers in arid regions. Researchers have posited its limited adoption to inadequate marketing and reduced appeal owing to its small seed size, prostrate plant habit, and culinary characteristics^10^. Recent tepary bean breeding efforts improved yield, adaptation, seed size, seed quality, common bacterial blight resistance, and rust resistance^11^, highlighting its potential for improvement via focused breeding efforts. Indeed, tepary bean could serve as an alternative pulse crop for marginal environments and as a genetic resource for abiotic stress tolerance to breed more resilient common beans under a changing climate.

In this work, we describe reference genome sequences for the cultivated landrace Frijol Bayo (*P. acutifolius* var. *acutifolius;* PI 692269; G40001-Seq) and the wild accession W6 15578 (*P. acutifolius* var. *acutifolius*; PI 638833). Gene expression responses of Frijol Bayo relative to a heat-tolerant common bean variety (Amadeus-77)^12^ under moderate night-time temperature stress reveal differential thermotolerance responses in tepary compared to the common bean. Landrace and wild gene family composition differ with those in common bean, with a special reference to the disease resistance genes associated with effector-driven resistance to pathogens. Loci associated with the domestication of *P. acutifolius* and *P. vulgaris* have limited overlap. These genome resources and associated analyses provide insights into innovations in adaptation to diverse environments and provide a roadmap for improvements of both *Phaseolus* species needed for the climate stress environments of the twenty-first century.

## Results

### De novo genome assembly and annotation

**Fig. 1:**
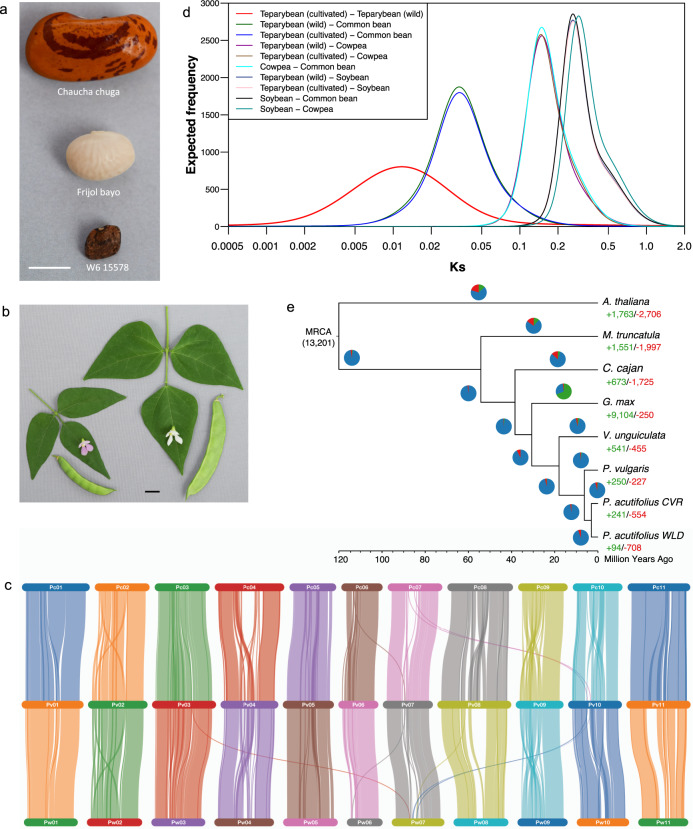
Genome evolution and divergence of common and tepary bean. **a** Seed diversity of sequenced beans. *Phaseolus vulgaris* Chaucha chuga (G19833, Andean landrace), *Phaseolus acutifolius* Frijol Bayo (G40001, cultivated landrace), and *P. acutifolius* W6 15578 (wild accession). The line represents 5 mm. **b** Trifoliolate leaves, flowers, and pods of W6 15578 (left) and Frijol Bayo (right). The line represents 10 mm. **c** Synteny between *P. vulgaris* G19833 (Pv; center) and wild W6 15578 (Pw; bottom) and cultivated Frijol Bayo (Pc, top) *P. acutifolius*. **d** Estimated divergence time between *P. acutifolius* (W6 15578 vs Frijol Bayo) and other legumes. **e** Expanded and contracted gene families were determined with CAFE and 13,201 families were inferred to be present in the most recent common ancestor (MCRA). The number of families that were expanded (green in the pie chart) or contracted (red in the pie chart) are plotted in a pie chart along with the number of neutral families (blue in pie chart) for each of the evolutionary divisions and species. The ultrametric input tree is displayed and the number of expanded and contracted families in each species is indicated below the species label. Source data underlying Fig. 1e is provided as a Source data file.

Frijol Bayo is an indeterminate, photoperiod insensitive, and broadly adapted white-seeded landrace used to introgress common bacterial blight resistance from tepary to common bean^13^ (Fig. 1a, b). W6 15578 is an indeterminate, photoperiod sensitive wild accession with small, mottled seeds, and pods that shatter at maturity and used to improve abiotic stress tolerance in common bean^8^. Reference genome assemblies for tepary bean were generated using Pacific Biosciences (PACBIO) long-read sequencing for Frijol Bayo with the MECAT assembler^14^, and Illumina paired-end, mate-pair, and linked reads with NRGene’s DeNovoMAGIC2^TM,^^15^ platform for W6 15578 and scaffolded into pseudochromosomes using proximity-by-ligation and anchoring to genetic maps (Table 1). Both assemblies provided robust representation of the gene space as reflected in the detection of 92.5% (Frijol Bayo) and 92.9% (W6 15578) of the BUSCO Embryophyta orthologs (Supplementary Data 1). Based on k-mer analysis, the estimated genome sizes for Frijol Bayo and W6 15578 are 684 Mb and 676 Mb, respectively (Supplementary Fig. 1), in agreement with flow cytometry which estimated both Frijol Bayo and W6 15578 at 682 Mb. Repetitive sequence content in Frijol Bayo was comparable to that of *P. vulgaris* G19833 (53.0% vs 55.1%, Supplementary Data 2) whereas W6 15578 had a higher repetitive sequence content (64.2%) than Frijol Bayo and *P. vulgaris* G19833; the majority of this increase was attributable to unclassified repetitive sequences. It is unlikely that the wild W6 15578 genome has substantially more repetitive sequences than the domesticated landrace Frijol Bayo. *P. vulgaris* G19833 and Frijol Bayo were sequenced and assembled with long read PACBIO reads while W6 15578 was assembled with linked short reads and a different assembler, thus these differences in genome size and repetitive sequence content may be technical in nature. The Frijol Bayo and W6 15578 genomes showed no large rearrangements or gaps outside of the heterochromatic, pericentromeric regions (Fig. 1c and Supplementary Figs. 2 and 3). Annotation of the Frijol Bayo and W6 15578 genomes resulted in a similar number of high-confidence genes for Frijol Bayo (27,538) and W6 15578 (27,095) and robust representation of the BUSCO orthologs with 94.6% and 95.7% complete BUSCO orthologs, respectively (Table 1 and Supplementary Data 1).

**Table 1:** Genome assembly and annotation metrics of *Phaseolus* species.

	*Phaseolus acutifolius* Frijol Bayo, Cultivated	*Phaseolus acutifolius* W6 15578, Wild	*Phaseolus vulgaris* Chaucha chuga v2.1^a^
Total assembly length	512,626,114 bp	661,870,301 bp	537,218,636 bp
Total contig number	667	29,934	1044
Contig N50 size (L50 number)	6,178,816 bp (25)	47,040 bp (3,492)	1,885,876 bp (73)
Total Scaffold number	155	609	478
Scaffold L50 size (N50 number)	45.2 Mb (5)	42.6 Mb (8)	49.7 Mb (5)
Percent assembly anchored to 11 pseudomolecules	99.00%	70.54%	95.67%
Number of high-confidence genes	27,538	27,095	27,433
Number of high-confidence gene models	50,635	40,833	36,995

^a^*Phaseolus vulgaris* Chaucha chuga v2.1 statistics were taken from Phytozome (https://phytozome.jgi.doe.gov/pz/portal.html#!info?alias=Org_Pvulgaris).

### Tepary bean is a sister species to the common bean

Nucleotide substitutions at synonymous sites (*Ks*) are selectively neutral^16^ and are used as a molecular clock to date the divergence time of closely related species. Individual peaks in *Ks* distributions of orthologous genes between two species follow a Gaussian (normal) distribution and can be detected using mixture model analysis. Synonymous substitutions between pairs of orthologous genes in cultivated (Frijol Bayo) and wild (W6 15578) tepary bean, and other legume crops, including *P. vulgaris* (common bean), *Glycine max* (soybean), and *Vigna unguiculata* (cowpea), were used to estimate the divergence time between these genomes. Mixture model analysis of *Ks* distribution of orthologous genes between common bean and soybean revealed a single peak with a mean *Ks* value of 0.32 (Fig. 1d and Supplementary Data 3). Using the widely reported age of divergence between common bean and soybean^17,18^ of ~19.2 Mya, and the mean *Ks* value of the corresponding peak (0.32), we extrapolated the rate of silent mutations for leguminous species to be 8.3 × 10^−9^ substitutions/synonymous site/year (see “Methods” section). The age of divergence between Frijol Bayo and W6 15578 was estimated to be 0.77 million years based on this calibrated synonymous substitution rate. The geometric mean of the peak observed in the *Ks* distribution (*Ks* = 0.013) derived from a mixture model analysis of *Ks* distributions revealed the presence of a major peak at *Ks* = 0.013 (Fig. 1d and Supplementary Data 3). Based on similar comparisons, the divergence of tepary bean (Frijol Bayol) from other warm-season legume crops, such as common bean, cowpea, and soybean, was estimated to have occurred 2.1, 10.1, and 19.1 Mya, respectively (Fig. 1d and Supplementary Data 3). Similarly, the ages of divergence between cowpea and common bean, and cowpea and soybean, were estimated to be 10.3 and 21.2 Mya, respectively.

Both Frijol Bayo and W6 15578 shared substantial orthology with *P. vulgaris* as reflected in the species tree and shared gene families (Fig. 1e and Supplementary Fig. 4). As expected, with a limited divergence time from *P. vulgaris*, gene family expansion and contraction frequency were low, 241 genes expanded and 554 contracted in Frijol Bayo relative to the cultivated *P. vulgaris* G19833 reference genome (Fig. 1e). Gene ontology enrichment of genes expanded in Frijol Bayo and W6 15578, relative to *P. vulgaris* G19833, included genes involved in chitin binding, chitinase activity, cell wall macromolecule catabolic/metabolic processes, and amino sugar metabolic processes (Supplementary Fig. 5).

Synteny analysis between *P. vulgaris* G19833 and the *P. acutifolius* Frijol Bayo and W6 15578 genomes revealed 403 collinear blocks ranging from 5 to 2,684 orthologous pairs with 90.7% of W6 15578 and 91.3% of the Frijol Bayo orthologous pairs present in collinear blocks with *P. vulgaris* (Fig. 1c). This high level of collinearity between *P. vulgaris* and *P. acutifolius* is likely due to the short divergence time of 2.1 Mya. Breaks in synteny relative to *P. vulgaris* G19833 include intrachromosomal rearrangements on chromosomes 2, 3, and 9 previously identified by Gujaria-Verma et al.^19^ as well as on most other chromosomes in the pericentromeric regions (Fig. 1c and Supplementary Fig. 3). Introgression between common and tepary bean is challenging with hybridization requiring embryo rescue and backcrossing due to hybrid sterility^13,20^. While we did not detect large-scale interchromosomal structural rearrangements, the small structural variations, as well as the larger internal rearrangements present on chromosomes 2 and 9 could contribute to difficulties in creating fertile hybrids^20^.

### Tolerance in desert-adapted tepary bean to moderate elevated temperature

Access to the tepary genome sequence will enable the discovery of genetic factors associated with climate resilience. While high night-time temperature is known to decrease crop yield in common bean when it occurs during micro- and megasporogenesis, tepary bean tolerates elevated temperature. To rule of possible reasons of differential heat tolerance, Frijol Bayo and Amadeus-77, a Middle American small red common bean which shows heat tolerance relative to other common beans, were exposed to moderate night-time temperature stress (32 °C/27 °C, day/night), and gene expression profiles were compared during the first 24 h (hrs) of night-time stress induction. Not only did the absolute numbers of differentially expressed genes differ between Frijol Bayo and Amadeus-77 across the time course, but the overall temporal pattern and magnitude of genes impacted differed between these sister species (Supplementary Fig. 6), suggesting substantial differences in gene regulatory responses to elevated temperature.

**Fig. 2:**
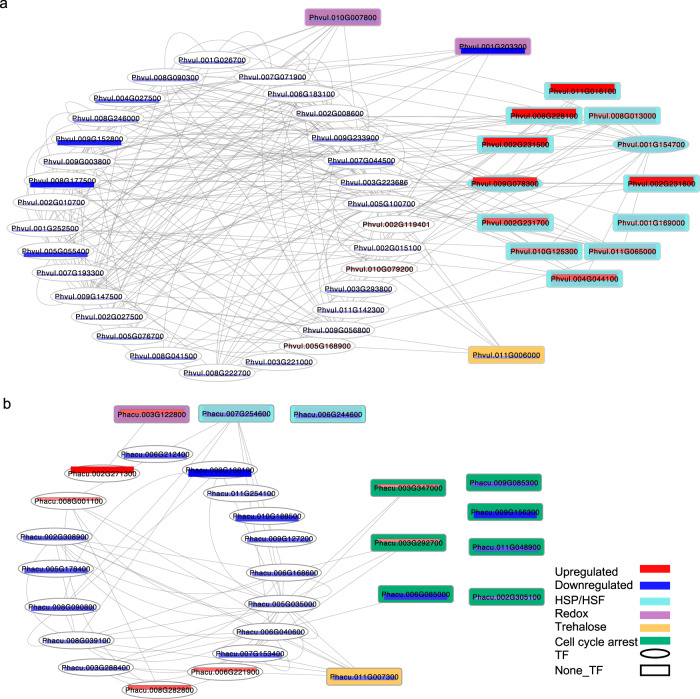
Response to moderate heat stress in tepary bean. Gene expression network showing different early responses to moderate night temperature (32 °C day/27 °C night) between *Phaseolus vulgaris* Amadeus-77 (**a**) and *Phaseolus acutifolius* Frijol Bayo (**b**). The network was created by ARACNeAP^95^. Transcription factors (TF) are within ovals and non-TF genes are within rectangles. Upregulation and downregulation of each gene is indicated by red and blue bars within the gene names. The width of the bars reflects the magnitude of the difference in relative expression values. Genes encoding heat shock proteins/heat shock factors (HSP/HSF), redox, trehalose, and cell cycle arrest are colored in aqua, lavender, goldenrod, and light green, respectively.

The heat shock response is evolutionarily conserved across all kingdoms of life, and entails a suite of physiological, molecular, and genomic responses that enable the repair of cellular machinery. Activation of transcriptional cascades following heat stress is common in a wide number of plant species as exemplified in *Arabidopsis thaliana* where 21 heat shock transcription factors (HSFs) and >2500 genes were differentially expressed following heat stress^21^. Within 1 h of the heat stress treatment, 15 genes encoding heat shock-related proteins (HSPs), including the master regulator HSFA2, were upregulated in Amadeus-77 while one was downregulated (Fig. 2a; Supplementary Figs. 7 and 8). Throughout the 24-h time course, upregulation rather than downregulation of HSPs was observed in Amadeus-77. This contrasted with Frijol Bayo, in which only three heat shock-related genes were downregulated at 1 hr. Even after 6 and 12 h of heat stress, the predominant response in Frijol Bayo was down- rather than upregulation of heat shock-related genes. The highest number of upregulated HSPs in Frijol Bayo was seven at 12 h after stress while the majority of genes from this family were downregulated. The substantial induction of the heat shock response in common but not tepary bean to an elevation of day- (32 °C) and night-time (27 °C) temperature is consistent with the evolution and adaptation of tepary bean to the hot Sonoran Desert environment. In a second heat stress experiment, exposure of Frijol Bayo to prolonged and elevated temperatures (36 °C day/32 °C night) in which leaves emerged and developed under heat stress conditions, upregulation of 15 HSPs was observed in tepary bean (Supplementary Data 4 and 5) suggesting that relative to common bean, tepary bean has an elevated temperature threshold for induction of the canonical heat shock response.

### Cell cycle and protectant molecules are induced following exposure to moderate heat stress

A moderate night-time temperature (27 °C) is sufficient to impact yield in common bean but not tepary bean. Interestingly, while Frijol Bayo did not respond with a canonical heat shock response following exposure to moderate heat stress (27 °C night-time temperature), it is clear Frijol Bayo perceived the heat stress within 1 h based on differential gene expression. We observed enrichment of gene ontology (GO) terms associated with phosphorelay signal transduction system (GO:0000160), negative regulation of cellular process (GO:0048523), cell cycle arrest (GO:0007050), and negative regulation of cell cycle (GO:0045786) (Fig. 2b and Supplementary Data 6,7). Eight differentially expressed cell cycle pathway genes were identified at 1 h in Frijol Bayo; two upregulated genes (*Phacu.CVR.003G292700* and *Phacu.CVR.003G347000*) were orthologs of the Arabidopsis *KIP-RELATED PROTEIN 7* (*KRP7 or ICK5*) which encode a CDK (cyclin-dependent kinase) inhibitor and negative regulator of cell division^22^ (Fig. 2b and Supplementary Fig. 9). Six orthologs of E2F target genes that control transcription of downstream cell cycle genes were downregulated at 1 h in Frijol Bayo including *Phacu.CVR.006G085000* (*CDC6A*, *CELL DIVISION CONTROL 6*), *Phacu.CVR.009G156300* (*CDT1a*), *Phacu.CVR.001G020700* (*ORC2, ORIGIN RECOGNITION COMPLEX 2*), *Phacu.CVR.002G305100* (*FAS2*), *Phacu.CVR.003G169400* (*MCM2, MINICHROMOSOME MAINTENANCE 2*), *Phacu.CVR.011G048900* (*MCM4*, *MINICHROMOSOME MAINTENANCE 4*), and *Phacu.CVR.009G085300* (*ETG1*, *E2F TARGET GENE 1*). This combination of up- and downregulated genes in Frijol Bayo is suggestive of a G1-S phase transition arrest in the cell cycle that may result when low-energy homeostasis induces *KIP-RELATED PROTEINs*^23^. During G1, the cell commits to cell division or exits the cell cycle. Such control enables the cells to respond to environmental cues, such as nutrition availability or abiotic stresses, by inhibiting cell growth and development in order to conserve energy^23^. By contrast, only one cell cycle gene *Phvul.011G017500*, an ortholog of *Arabidopsis Recognition Complex subunit 3* (*ORC3*) that initiates DNA replication at S phase, was upregulated in Amadeus-77 at 1 h.

At 3 h after stress induction in Frijol Bayo, GO terms related to cell redox homeostasis (GO:0045454), cellular homeostasis (GO:0019725), and homeostatic process (GO:0042592) were enriched (Supplementary Data 7) including seven genes from the glutaredoxin family (GRXs) and two genes from the thioredoxin (TRXs) family; all were upregulated. Glutaredoxins (GRXs) are small ubiquitous glutathione (GSH)-dependent oxidoreductases involved in oxidative stress responses^24,25^. All of the differentially expressed GRXs are of the CC-type. This included a homolog (*Phacu.CVR.011G148000*) of Arabidopsis GRXC9 that is induced by salicylic acid (SA) and is part of the early SA-induced non-expressor of pathogenesis-related (PR) genes 1 (NPR1)-independent pathway in which GRXC9/ ROXY19 interacts with class II TGA transcription factors to induce genes with antioxidant and detoxifying activities^26^. In Arabidopsis, 10 other CC-type GRXs also interact with TGAs. Among these are ROXY 10, and Roxy 21 (moderate effect) for which we found homologs in Frijol Bayo^27^. Redox genes that were upregulated at 6 h and peaked at 12 h after stress induction included four genes continuously upregulated from 3 to 12 h after stress induction; these genes were homologs of Arabidopsis *ACHT4*, *ROXY 21*, and *ROXY 10*.

Four trehalose synthesis-related genes were upregulated at 3 h of heat stress in Frijol Bayo: *Phacu.CVR.003G017200* [homolog of Arabidopsis *TREHALOSE -6-PHOSPHATASE SYNTHASE* (*TPS6*), *Phacu.CVR.003G183300* (homolog of Arabidopsis *TPS11*), *Phacu.CVR.009G053300*, and *Phacu.CVR.002G288900* (both homologs to Arabidopsis *TREHALOSE-6-PHOSPHATE PHOSPHATASE J* (*TPPJ*))]. In contrast, trehalose biosynthesis genes in Amadeus-77 [*Phvul.002G102300* (*TPS8*, trehalose-6-phosphate synthase 8), *Phvul.009G180300* (*TPS9*), *Phvul.003G053000* (*TPS10)*, and *Phvul.003G150400* (*TPS11*)] were downregulated at 1 h post-stress. Previous studies have demonstrated that high concentrations of trehalose protect cell membranes and proteins from degradation resulting from heat, oxidative stress, drought, and frost stress^28–34^, thereby enabling organisms with high trehalose concentration (including some plants) to survive the stress by remaining in a quiescent state^35^. The response in tepary bean is consistent with a number of plant species where trehalose-6-phosphate synthase is overexpressed under drought and heat stress^35,36^ and in desiccation-tolerant species that survive in extreme habitats^35,37^. In Arabidopsis, trehalose accumulates in response to heat stress and trehalose biosynthesis mutants had reduced survival rates following heat stress^38^. Furthermore, supplemental trehalose can rescue tolerance to heat stress^38^ and overexpression of a chimeric yeast translational fusion of trehalose-6-phosphate synthase and trehalose-6-phosphate phosphatase resulted in heat tolerance, including tolerance to temperatures of 56 °C for 3 h with no pre-acclimation period^39^. Examination of the transcriptional coactivator *MBF1c* (multiprotein bridging factor 1c) in Arabidopsis revealed that it functions upstream of trehalose in basal thermotolerance^38^. In Frijol Bayo but not Amadeus-77, *MBF1c* is upregulated at 1 and 12 h of heat stress consistent with MBF1c-mediated trehalose mechanism of basal thermotolerance in tepary bean. The levels of trehalose in vivo are usually too low to play a role as an osmoprotectant^40,41^ and there is evidence that trehalose-6-phosphate is a signal of sucrose status and, therefore, may act instead as a regulatory molecule for osmoprotection^36^. Future functional studies that measure the cell cycle and synthesis of redox and trehalose in tepary and common bean following heat stress will further our understanding of the physiological and biochemical mechanisms of thermotolerance in *Phaseolus*.

### Expansion of the NB-ARC gene family in *Phaseolus vulgaris*

Plants evolved a modular-domain protein family as a critical component of resistance against biotic pathogens. Central to this family is a canonical NB-ARC domain bordered by C-terminal leucine-rich repeat domains (NLR proteins), and often an N-terminal coiled-coil or TIR domain. Biotic resistance can be activated when a pathogen-specific effector or an effector/host protein complex molecule interacts with one of the canonical domains. Furthermore, other NLR proteins contain a non-canonical integrated domain (ID) that interacts with the effector^42,43^. The implication of this discovery is that a much greater repertoire of domains than the canonical NLR domains, can sense the presence of an effector in the plant cell. Given that tepary originated from an arid environment, and that biotic pathogens often favor humid conditions to thrive, a comparison of the NB-ARC repertoire in tepary and common bean, a temperate sister species that have higher water requirements for growth, may shed light on the evolution of this protein family under an abiotic stress environment.

**Fig. 3:**
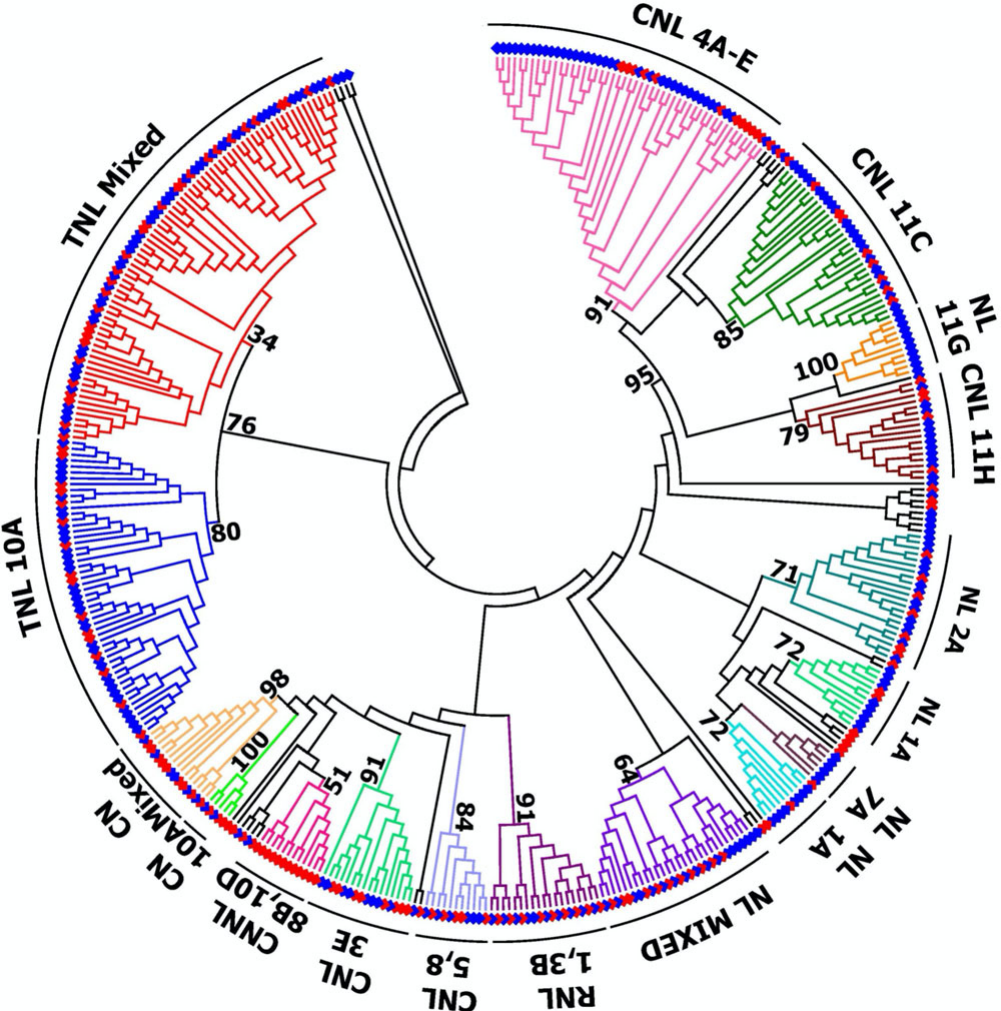
Phylogeny of the *Phaseolus acutifolius* Frijol Bayo and *Phaseolus vulgaris* G19833 NB-ARC gene families. Neighbor-joining tree of the NB-ARC containing gene models from the two species. Cluster designations (outer ring) are as described in Supplementary Data 8 and 9. Red diamonds represent *P. acutifolius* gene models, and blue diamonds represent *P. vulgaris* gene models. Bootstrap values (out of 100 replications) are shown for major nodes.

Because of the importance of the NB-ARC domain in activating the defense response, genome-wide searches for potential NLR resistance genes are based on the presence of this domain. The Frijol Bayo genome has 200 genes with an NB-ARC domain (Supplementary Data 8 and Fig. 3), of which 155 encode NLR proteins. By comparison, the common bean genome (Chaucha Chuga, G19833) encodes 330 NB-ARC domain proteins (Supplementary Data 9), and of these, 248 encode NLR proteins. To assess whether the sizes of tepary and common bean NB-ARC families evolved differently than other gene families, we compared the size of 80 transcription factor (TF) families in these two genomes. The total number of TFs across families in tepary (*n* = 1818; Supplementary Data 10) and common bean (*n* = 1881; Supplementary Data 11) were similar, and a high correlation (*r* = 0.99) was observed for the number of members of the 80 transcription factor families between the two genomes (Supplementary Data 12). Therefore, unlike the size of transcription factor families which are conserved within the *Phaseolus* genera, the NB-ARC family differs in size between these two *Phaseolus* species suggesting unique evolutionary dynamics for this gene family.

Multiple domain structures are possible because of the modular nature of NB-ARC proteins^44^. The Frijol Bayo genome encodes 20 different domain structures, while the G19833 common bean genome encodes 23 (Supplementary Data 13). The primary difference between the two genomes is the greater number of common bean proteins encoding an NB-ARC/LRR domain structure. For Frijol Bayo, 34 NB-ARC genes encode proteins with an ID, compared to 40 in common bean (Supplementary Data 14). Two of the tepary and common bean IDs, zf-BED (Pf02892) and zf-RVT (Pf13966), are frequently found in NLR genes from other species, and were noted in a previous assembly of common bean^45^. Of the 58 IDs identified collectively between tepary and common bean, only eight were shared between the two species. This points to the unique evolution of tepary NB-ARC genes relative to the common bean. Further, of the 58 IDs found in *Phaseolus* spp., only nine were integrated into NB-ARC proteins in other plant genomes. This discovery supports previous observations that many IDs are associated with a large array of cellular functions that have fused with NB-ARC domains to develop potential sensors of pathogen effectors^45,46^.

Early genetic experiments demonstrated that resistance genes are tightly linked within the genome^47^, while subsequent research in many plant species demonstrated that physical clusters of NB-ARC genes often contain functional NLR genes^48^. Clustering of NLR genes is also a feature of the *Phaseolus* genus where a total of 21 clusters were observed in tepary and 25 in common bean with 63% and 73%, respectively, of the NB-ARC genes located in clusters (Supplementary Data 15). In most cases, each tepary NB-ARC cluster has a corresponding cluster in common bean with genes of similar domain structure: IA = NL; 2B = TNL; 3A = RNL. While some of the clusters are of equal size in the two genomes (2B, 3A), other clusters (4A, 10A, 11C) have greatly expanded in common bean relative to tepary bean. As observed previously for common bean chromosomes Pv04^49^ and Pv11^50^, many clusters are located on the ends of the chromosomes. The only exceptions are NL clusters 2A and 10D that are located in the recombination poor heterochromatic region. For common bean, many genetically mapped resistance factors are associated within clusters^51^, yet the only functionally proven R gene in common bean with a NB-ARC domain (*Phvul.005G031200*) resides as a singleton^52^.

Phylogenetic analyses uncovered several evolutionary patterns in the NB-ARC gene families in the Frijol Bayo and G19833 genomes (Fig. 3). Many of the Frijol Bayo genes (Fig. 3; red diamonds) are paired with a G19833 ortholog. Most of these are also best reciprocal BLASTP hits (Supplementary Data 16), especially those with high identity between the two orthologs. As often seen in plant species, the TNL and non-TNL NB-ARC genes form two distinct phylogenetic clusters. The Pv04 and Pv11 CNL clusters group together at a higher taxonomic level suggesting an ancestral relationship. Two examples of NB-ARC gene expansion in Frijol Bayo were noted, clusters 8B and 10B with the NNL or CNNL domain structure, and five genes (between the NL 1A and NL 7A) that have short (<88 amino acids) NB-ARC sequences. The greatest expansion in G19833 occurred at the distal ends of Pv04 and Pv11, respectively. The first 21 common bean NB-ARC genes in the CNL4A-E group are specific to cluster 4A. The same Frijol Bayo cluster only consists of six NB-ARC genes. On Pv11, clusters 11G and 11H are entirely (11G) or almost entirely (11H) represented by G19833 genes at the distal end of the long arm of Pv11. This expansion supports the suggestion that the ends of chromosomes are hot spots for resistance gene evolution via expansion^50^. Contrary to these clusters, the RNL cluster 3B is nearly a perfect one-for-one match between the two species.

### Population structure, domestication, and adaptation

**Fig. 4:**
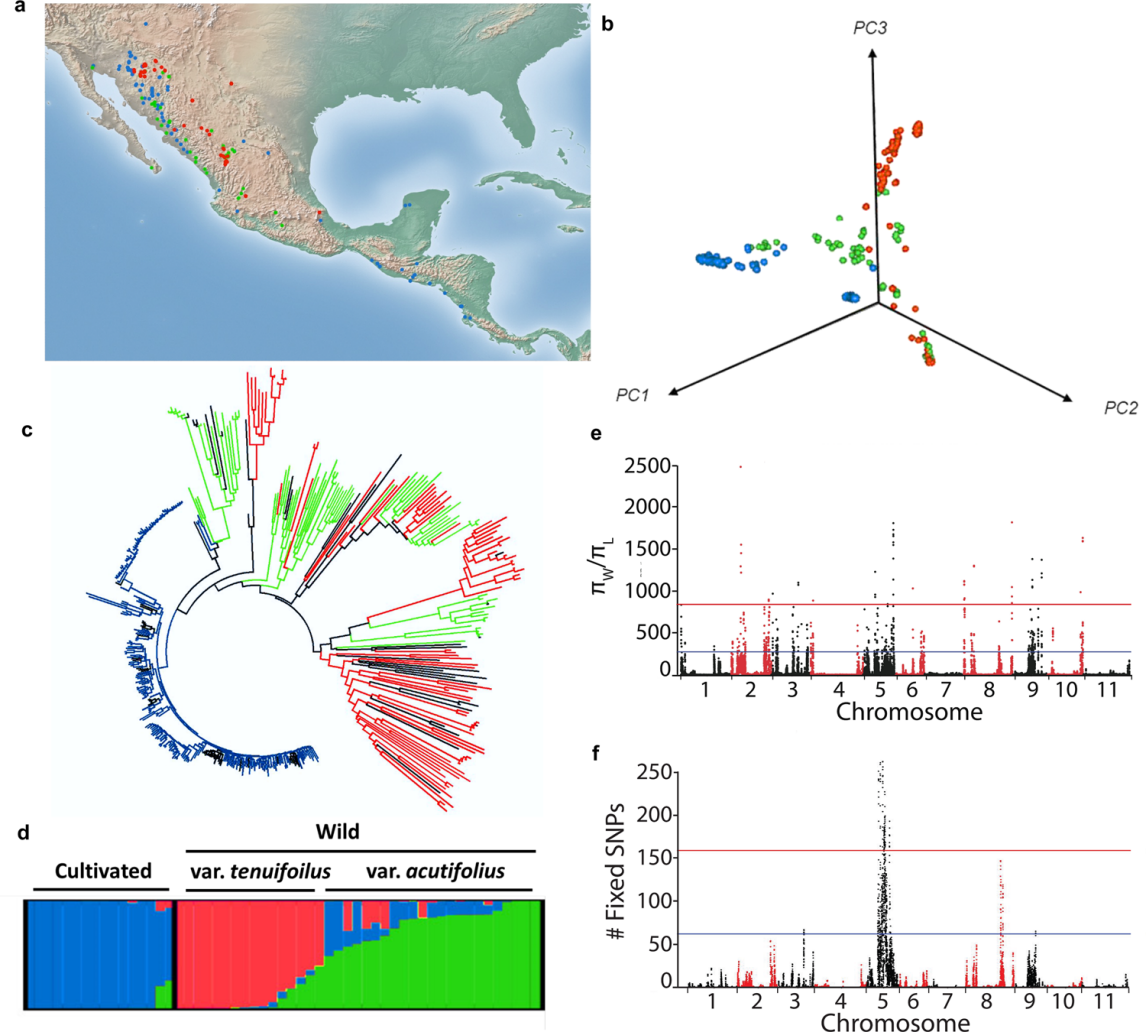
Population genomics of tepary bean. Geographic distribution (**a**), PCA analysis (**b**), neighbor-joining tree (**c**), and structure analysis (**d**) of the wild and cultivated tepary beans. Cultivated: blue, Wild: red (*P. acutifolius* var. *tenuifolius*), green (*P. acutifolius* var. *acutifolius*), Unknown: black. **e** π ratio distribution over 100k–10 kb windows across tepary genome revealed the regions that underwent reduction or complete loss of diversity; alternate red and black colors are used to differentiate the 11 chromosomes. **f** Distribution of SNPs in which one of the allele presents in wild but another allele presents in landraces revealed that Pc05 is involved the most in the adaptation process of tepary; alternate red and black colors are used to differentiate the 11 chromosomes.

A total of 28,750 physically mapped single nucleotide polymorphisms (SNPs; MAF ≥ 0.05) were identified among 425 wild, weedy, and cultivated tepary genotypes. These represent the Tepary Diversity Panel, a collection of genotypes representing the entire geographic distribution of the wild and cultivated tepary beans (Fig. 4a). A principal component analysis (PCA) using this variant data set (Fig. 4b) showed that much of the variation was accounted for by PC1 (25.6%) while the first three PCs account for 45.2% of the variation. The neighbor-joining (NJ) tree analysis with *n* = 2317 SNPs with LD values <0.1 clearly separated the cultivated and wild/weedy genotypes (Fig. 4c). Cultivated genotypes from two geographically distinct regions, the Sonoran Desert region and the Pacific Coast of Central America, were closely related phylogenetically. Climatically, these two regions experience high, dry desert heat (Sonora) or high, humid coastal heat (Central American Pacific coast) suggesting that within the narrow diversity within the cultivated population, genetic variability exists for adaptation to these two distinct heat stress environments.

A subset of 55 geographically diverse genotypes representing the geographical range in the Tepary Diversity Panel was selected for whole-genome resequencing (Supplementary Data 17). A STRUCTURE analysis suggested three subpopulations (Fig. 4d). The narrow genetic base of cultivated tepary was best demonstrated by the observation that among 3,377,439 SNPs, 2,247,877 (66.6%) SNPs were polymorphic among the wild yet monomorphic among the cultivated genotypes (Supplementary Table 1). Overall genes, 5003 (16%) were monomorphic among both wild and cultivated genotypes.

Population genomic analyses revealed signatures of domestication and adaptation in tepary bean. As demonstrated in the phylogenetic analyses, the wild population was more diverse [π (per bp) = 0.0018; Ɵ = 0.0013] than the cultivated population [π (per bp) = 0.0007; Ɵ = 0.0005]. Domestication signatures were identified^16^ using the *π*_wild_/*π*_landrace_ ratio over 100 kb/10 kb sliding windows (Fig. 4e) and focusing on the windows in the 0.1% and 1.0% tails of the bootstrap distribution. Chromosomes Pa07 and Pa11 were essentially monomorphic between the wild and cultivated populations, while the two populations were highly polymorphic for chromosome Pa05. Chromosomes Pa02 and Pa03 were polymorphic across the euchromatic tails, and the remaining chromosomes exhibited high π ratios isolated to narrow genomic regions. A total of 100 genomic blocks, consisting of neighboring windows in the 0.1% bootstrap tail, contained 591 genes. A reciprocal BLASTP analysis of the 591 tepary domestication genes found that only 32 were homologs of the common bean Middle American domestication genes (*n* = 1835^18^), and four were homologs of the common bean Andean domestication genes (*n* = 748^18^). The comparison found 20 of the tepary/Middle American shared domestication genes were located on chromosome nine of each genome. All 20 of these shared homologs were all located in shared domestication blocks of the two genomes. Overall, this demonstrates that domestication involved unique sets of genes in each of these two *Phaseolus* genomes.

The total length of the cultivated tepary genome blocks with low diversity, and presumed to be associated with domestication, was 8.50 Mb. Furthermore, a total of 242 out of 591 genes located within these domestication blocks were monomorphic relative to their wild orthologs (Supplementary Data 18). Collectively, those blocks containing monomorphic genes covered 1.75 Mb. Compared with the *P. vulgaris* G19833 genome, a greater proportion of the wild tepary bean genome became monomorphic during domestication. Gene ontology enrichment analyses of the 242 genes with low diversity among cultivated genotypes identified genes associated with ion transport enriched (Supplementary Fig. 10). Ion transport regulators were also associated with the domestication of salt-tolerant weedy rice^53^ and maize^54^.

Adaptation occurs when individuals from the initial domestication environment are grown in other environments and diversity is selected from standing variation or occurs via de novo mutation. To identify such regions, SNPs in which one allele was fixed in all wild genotypes and the alternate allele was fixed in all landraces were investigated. All of the fixed SNPs in the 0.1% bootstrap tail were located on Pa05 while the second region of fixed SNPs in the 1% tail was located on Pa08 (Fig. 4f). Of the 98 Pa05 genes associated with these fixed SNPs, GO enrichment revealed an association with inositol phosphatase activity (Supplementary Fig. 11). The role of inositol phosphatase in plant adaptation under abiotic stresses is well documented^55^. Inositol phosphatase regulates/catalyzes the network of inositol phosphate and phosphoinositide signaling pathways which results in altering other stress response pathways including calcium release and abscisic acid signaling which have important roles in plant growth in stressful environments.

## Discussion

Tepary bean is an orphan crop distinguished from other pulse crops by its abiotic stress tolerance, primarily thought to result from its origin and evolution in the hot and dry Sonoran Desert. Access to the genome of tepary bean revealed exceptional synteny and orthology with common bean yet highlighted mechanisms of resilience. Under a moderate night-time heat temperature which is limiting to yield in common bean but not tepary bean, gene expression analyses suggest that tepary bean arrests the cell cycle and synthesizes compounds with redox potential as well as the disaccharide trehalose that has been associated with abiotic stress tolerance. However, tepary bean is not immune to the impacts of heat stress, and exposure of tepary bean to prolonged and elevated night-time heat stress resulted in upregulation of genes encoding the canonical heat shock response. Evolution in arid conditions most likely resulted in reduced biotic pressure and as a consequence, the classical disease resistance genes family evolved differentially in common and tepary bean.

Currently, the common bean is the most important legume source of plant protein for direct human consumption. However, common bean production is increasingly limited geographically due to its sensitivity to high temperatures and its susceptibility to diseases due to climate change. In fact, it is predicted that by 2050, not only will the majority of common bean production areas in southeastern Africa be unsuitable, but the nutritional content of common bean will also be reduced^56^. Interestingly, the protein and nutritional composition of tepary are similar to its sister species, *P. vulgaris*^57^. The close sister-species relationship between *P. vulgaris* and *P. acutifolius* provides a powerful framework for the improvement of tepary agronomic and disease resistance traits through introgression from common bean, and the improvement of common bean abiotic stress tolerance via introgressions from tepary bean. For example, work nearly a century ago in common bean showed that the presence of recessive alleles at the *Fin* locus conferred determinate growth habit^58^. *Fin* encodes an ortholog of the *Arabidopsis thaliana* TERMINAL FLOWER 1 (*PvTFL1y*) for which known mutations lead to a determinate growth habit^59,60^. *Phacu.001G234100*, a *P. acutifolius* homolog of *PvTFL1y*, is 99% identical to the *P. vulgaris* indeterminate allele, providing a target to alter the growth habit of tepary via mutagenesis or gene editing. Tepary bean also provides a resource for allele and trait mining across the *Phaseolus* genus as exemplified by the *P* (*Pigment*) locus, a basic helix-loop-helix (bHLH) transcription factor, shown to control seed coat color in common bean^61^. In white-seeded Frijol Bayo, a ~56 kb inversion involving six genes disrupts the putative tepary *P* gene whereas the dark-seeded W6 15578 *P* ortholog lacks this inversion (Supplementary Fig. 12). Given the expansion of crop production into areas marginalized by warming and drying climates, leveraging knowledge and genetic resources and applying comparative genomics across these *Phaseolus* species provides a powerful approach to accelerate the improvement of climate-adapted bean varieties to address climate change.

## Methods

### Germplasm description

Frijol Bayo, was collected in a market in Cordoba, Veracruz, Mexico in 1951 by Howard Scott Gentry. It is a white-seeded cultivated tepary bean that is photoperiod insensitive, has an indeterminate growth habit, has a hundred seed weight of 12.3 g, and exhibits broad adaptation. Frijol Bayo shows resistance to common bacterial blight^62^ caused by *Xanthomonas axonopodis*, is drought tolerant^63^, heat tolerant^62^, and is resistant to leaf hoppers (*Empoasca krameri*)^13^. Frijol Bayo, and another accession, G40020, were used as parents in the early and challenging interspecific introgression of common bacterial blight resistance from tepary bean to common bean^13^ using embryo rescue, which subsequently resulted in the release of the VAX 1 to VAX 6 lines^6^. This broad and durable common bacterial blight resistance has been extensively introgressed into both common bean gene pools, aided by the use of the SU91 and BC420 markers^64^. A core recombinant inbred line population was recently developed from the cross Frijol Bayo and G40022. These lines facilitate the investigation of the genetic architecture of seed traits, agronomic characteristics, and disease response in tepary bean^62^.

Purification of the Frijol Bayo was completed over three generations of single-seed descent in a screenhouse at the USDA-ARS-TARS in Mayaguez, Puerto Rico, from 2015 to 2017. The original G40001 accession was received from the International Center for Tropical Agriculture Research. The purified G40001 seed lot was further increased in a screenhouse in the Fall of 2019. A total of 5000 bulked seed from the 2019 increase were provided to the USDA GRIN and assigned a unique accession name and number (G40001-Seq; PI 692269; https://www.ars-grin.gov/) to differentiate it from the original accession. Purified G40001 were dark adapted on two occasions in a dark, air-conditioned room for one day, and young trifoliolate leaf tissue was then harvested and immediately frozen in liquid nitrogen, and subsequently stored at −80 °C for high molecular weight DNA extraction at the University of Arizona.

W6 15578 is a wild *P. acutifolius* accession obtained from the Western Regional Plant Introduction Station of the USDA. Originally collected by O.W. Norvell in Mexico in 1955, it now has the Plant Introduction number PI 638833 (https://www.ars-grin.gov/). It is photoperiod sensitive, has an indeterminate growth habit, and has small, mottled seeds, and pods shatter at maturity. It was used as a donor parent in the development of an interspecific hybrid population to improve abiotic stress tolerance in common bean^8^. W6 15578 was one of the parents, along with PI 430219, of the tepary bean mapping population, BR-06, developed and genotyped at the University of Saskatchewan^19^.

### Genome sequencing and assembly

*P. acutifolius* Frijol Bayo was sequenced using a whole-genome shotgun sequencing strategy and standard sequencing protocols. Illumina fragment reads were generated using the Illumina X10 and HiSeq platforms and PACBIO reads were sequenced using the RS platform using Sequel® Sequencing Kit 2.1 v2. One 480 bp insert Illumina WGS library (159.2×), and one Dovetail Hi-C library (122.3×) was sequenced to 150 nt in paired-end mode (Supplementary Table 2). Prior to analysis, Illumina reads were screened for mitochondria, chloroplast, and PhiX contamination, and reads composed of >95% simple sequence were removed. Illumina reads shorter than 50 nt after trimming for adapter and quality (*q* < 20) were removed. For PACBIO sequencing, a total of 18 SMRT Cells (10-h movie time) were sequenced with a p-read yield of 55.7 Gb, with total coverage of 101.28× (Supplementary Table 3). The final read set consisted of 540,535,272 Illumina WGS reads (159.2× coverage) and 415,119,662 Hi-C reads (122.3x coverage).

The Frijol Bayo assembly was generated by assembling the 6,524,706 PACBIO reads (101.28× sequence coverage) using the MECAT assembler (v1.0)^14^ and subsequent polishing with ARROW (v2.2.2)^65^. V1 of the G40001 assembly was comprised of 1,092 scaffolds (1092 contigs), with a contig N50 of 6.2 Mb and 250 scaffolds larger than 100 kb representing a total genome size of 523.1 Mb (Supplementary Table 4). Misjoins in the assembly were identified using a combination of 529 genetic markers^19^, *P. vulgaris* synteny (version 2.1 release), and Hi-C scaffolding using the JUICER pipeline (v 1.5.6)^66^. A total of 16 misjoins were identified and resolved. Scaffolds were then oriented, ordered, and joined together into 11 chromosomes using the markers provided. A total of 512 joins were made during this process (Supplementary Fig. 13). Each chromosome join is padded with 10,000 Ns. The significant telomeric sequence was identified using the (TTTAGGG)_*n*_ repeat, and care was taken to ensure proper orientation in the production assembly. The remaining scaffolds were screened against bacterial proteins, organelle sequences, and GenBank nr, then contaminants were removed. Finally, homozygous SNPs and INDELs were corrected in the release consensus sequence using ~100× Illumina WGS reads (150 nt paired end, 480 bp insert) by aligning the reads using BWA-MEM (v 0.7.15)^67^ and identifying homozygous SNPs and INDELs with the GATK’s UnifiedGenotyper tool^68^ (v3.6-0-g89b7209); a total of 149 homozygous SNPs and 10,468 homozygous INDELs were corrected in the release. Frijol Bayo scaffolds that were not anchored on a chromosome were classified into bins depending on sequence content. Contamination was identified using blastn against the NCBI nucleotide collection (NR/NT) and blastx using a set of known microbial proteins. Additional scaffolds were classified as unanchored repeats (>95% masked with 24mers that occur more than four times in the genome) (422 scaffolds, 14.9 Mb), and mitochondria (eight scaffolds, 583.6 kb). The resulting final statistics are shown in Supplementary Table 5. The final version 1.0 release represents 512 Mb of sequence, consisting of 667 contigs with a contig N50 of 6.2 Mb and a total of 98.9% of assembled bases in chromosomes.

Wild tepary W6 15578 was selfed for several generations, and tissue was collected from a single plant for nuclear DNA isolation. Nuclei were isolated using the method developed by Zhang et al.^69^. High molecular weight (HMW) DNA was extracted from the nuclei using a modified CTAB extraction method^70^ at the University of Saskatchewan. A sample of the HMW DNA was sent to the University of Illinois Roy J. Carver Biotechnology Center for paired-end and mate-pair library construction following the same protocol as Chen et al.^15^ (Supplementary Table 6). A 10× Genomics^TM^ Chromium library was generated and sequenced to a depth of 175× at HudsonAlpha Institute for Biotechnology, Huntsville, AL. De novo genome assembly was carried out using the DeNovoMAGIC2 software (v2) platform^15^. The Chromium 10× data were used to support scaffold validation and elongate phased scaffolds (Supplementary Table 6). W6 15578 pseudomolecules were constructed by aligning flanking sequences from Illumina GoldenGate OPA and GBS genetic markers from two tepary genetic maps^19,71^ to the scaffolds using BWA (v0.7.1)^67^; only unique hits to the scaffolds were used for order and orientation. Where only a single marker was present, the scaffold was oriented according to synteny with common bean (Supplementary Fig. 14). Alignments of the 10× Chromium data to the scaffolds were also used to correct two orientation errors introduced by the maps. Reads from a single Oxford Nanopore library were used to confirm order where discrepancies occurred with the order of the cultivated assembly. Final assembly statistics are in Supplementary Table 7.

### Genome size estimates and quality assessments

Processed Illumina short-read data from Frijol Bayo and W6 15578 were provided to Jellyfish v2.2.6^72^ to generate k-mer (*K* = 21) frequency distribution with –C –m 21 –min-quality = 25 parameters. The output histograms were analyzed by findGSE (v0.1.0)^73^ to estimate the genome size, heterozygosity, and repeat fraction. The presence of conserved plant orthologs was evaluated using BUSCO^74^ (v3.0.2b) with Embryophyta database 9 and an *E*-value of 1*e*−05.

### Genome annotation

To annotate the Frijol Bayo genome, species-specific custom repeat library (CRL) was created using RepeatModeler (v1.0.8; http://repeatmasker.org), and putative protein-coding genes identified as repeats by RepeatModeler were identified and excised from the CRL by searching against a curated library of plant protein-coding gene sequences using ProtExcluder^75^ (v1.1). Output from ProtExcluder was then combined with the RepBase^76^ (v20150807) Viridiplantae repeats to create a final CRL and the final pseudomolecules were masked using RepeatMasker (v4.0.6; http://repeatmasker.org) with the CRL using the options: -s -nolow -no_is -gff. Reads from each RNA-seq library (see below) were adapter trimmed and quality filtered using Cutadapt^77^ (v1.18) with the options -n 2 -m 100 -q 10. Reads were then aligned to the assembly using HISAT2^78^ (v2.1.0) using the strand-specific mode and a maximum intron length of 5 kb and genome-guided transcript assemblies created from each alignment using Trinity^79^ (v2.6.6) with a minimum contig length of 500 bp and a maximum intron length of 5 kb.

Frijol Bayo gene models were predicted by first training Augustus^80^ (v3.2.2) with alignments of the mature leaf RNA-seq library to the soft-masked genome assembly, then running Augustus on the hard-masked genome. Gene models were then refined with PASA2^81^ (v2.2.0; https://pasapipeline.github.io/) using the genome-guided transcript assemblies as transcript evidence to produce the working set of gene models. To generate a high-confidence set of gene models, gene expression abundances of the working gene models for each RNA-seq library (see below) were generated using Kallisto^82^ (v0.45.0), and gene models were searched against PFAM^83^ (v32) using HMMER^84^ (v3.1b2). Gene models containing a PFAM hit and/or expression evidence in at least one RNA-seq library were defined as high-confidence models. Functional annotation was generated by searching the gene models against the *A. thaliana* proteome (TAIR10^85^), Swiss-Prot (2015_08)^86^, and PFAM^83^ (v32), and assigned a functional description. InterProScan (v. 5.34–73.0) was used to search the Frijol Bayo proteome using “goterms” option to assign locus gene ontology (GO). The GO terms assigned to *P. vulgaris* V2.1^18^ by Phytozome (https://phytozome.jgi.doe.gov/pz/portal.html) were used for common bean.

The W6 15578 genome was annotated using the MAKER-P (v2.31.10)^75^ annotation pipeline on the soft-masked genome sequence. RNA-seq (107 Gb) data from Frijol Bayo were pre-processed using Trimmomatic (v0.38)^87^ with the parameters: ILLUMINACLIP:2:30:10 LEADING:20 TRAILING:20 SLIDINGWINDOW:4:15 MINLEN:50. Processed RNA-seq reads were aligned against the W6 15578 genome assembly using STAR (v2.7.1)^88^; max. 3% mi-matches over 95% read length (max. intron size=10000), and assembled using Trinity^79^ (v2.8.4) genome-guided approach with the –genome_guided_max_intron 10000 option. The assembled Frijol Bayo transcripts, *P. vulgaris* proteins (V2.1; https://phytozome.jgi.doe.gov/pz/portal.html#!info?alias=Org_Pvulgaris), and the ab initio predictors (SNAP and Augustus, configured in hint-based mode) were used to generate initial W6 15578 gene models. The output from MAKER-P^75^ was processed by PASA^81^ (v2.3.3) to further incorporate the transcript alignment evidence into the initial gene annotation. High-confidence gene models were identified as described for Frijol Bayo above.

### Gene expression analyses

To generate a developmental gene atlas, seeds of Frijol Bayo were surface sterilized using 2% sodium hypochlorite for 10 min, rinsed, and soaked in Petri dishes at 29 °C/20 °C day/night temperature for 24 h and then planted in SUREMIX (SURE, Galesburg, MI, USA) in gallon size pots. Plants were grown under a 12 h photoperiod, 60% relative humidity, 29 °C day/20 °C night temperature (ramping up to maximum temperature and ramping down to minimum temperature over 1 h) in a controlled growth chamber with light intensity reaching full capacity of 500 μmol m^−2^ s^−1^ over one hour and dimming to complete darkness over one hour to mimic sunrise and sunset, respectively. A total of 12 developmental tissues were collected with sampling occurring between 10:00 and 11:30 am to minimize variation due to circadian rhythms (Supplementary Data 19). Each biological replication consisted of a pool of samples from three plants with three biological replications for each developmental stage. Total RNA was extracted from all tissues using the Spectrum™ Plant Total RNA Kit (Sigma-Aldrich, St. Louis, MO), and quality assessed using a Qubit fluorometer (Invitrogen INC. Carlsbad, CA) and Bioanalyzer 2100 (Agilent Technologies, Santa Clara, CA). Libraries were prepared using the KAPA RNA HyperPrep Kit (KK8540; KAPA Biosystems, Roche Sequencing Solutions, Pleasanton, CA, USA) using NEBNext oligos. Sequencing was performed on an Illumina HiSeq 4000; one biological replicate of all 12 tissues was sequenced using the 150 nt paired-end read configuration to achieve ~50 million read pairs for use in genome annotation. The other two biological replicates were sequenced to 50 nt in single-end configuration to generate greater than 25–30 million reads.

To determine expression abundances in the Frijol Bayo gene atlas, raw RNA-seq read quality was assessed using FastQC (v 0.11.5; https://www.bioinformatics.babraham.ac.uk/projects/fastqc/) and adapters and low-quality bases were removed using Cutadapt^77^ (v 1.16) with the following parameters:-q 20 -n 2 –trim-n –minimum-length 31. For expression abundance estimations, paired-end reads were trimmed to 50 nt, and all samples were subsampled at 20 million reads using seqtk (v 1.0.r82) (https://github.com/lh3/seqtk). Reads from all three replicates were aligned to the Frijol Bayo reference genome using HISAT2^89^ (v 2.1.0). Expression abundances in the form of Fragments per Kilobase of transcript per Million mapped reads (FPKM) were quantified using Cufflinks^90^ (v 2.2.1). Pairwise Pearson’s correlation was calculated for all samples using the “cor” function in R (v3.51; https://www.r-project.org/). *P*-values were calculated using R package “psych”. Principal Component Analysis (PCA) was conducted using the “prcomp” function in R (v3.51; Supplementary Fig. 15).

To understand gene expression under moderate heat stress, Frijol Bayo and Adamdeus-77 seeds were surface sterilized using 2% sodium hypochlorite for 10 min, rinsed, and soaked in Petri dishes at 29 °C/20 °C day/night temperature for 24 h (Frijol Bayo) or 72 h (Amadeus-77), and then planted in SUREMIX (SURE, Galesburg, MI, USA) in 4 × 4 × 6 inch pots in a growth chamber. Plants were grown under controlled growth chamber conditions with a 12 h photoperiod, 60% relative humidity, 29 °C day/20 °C night temperature for control, and 32 °C day/27 °C night temperature for heat stress (ramping up to maximum temperature and ramping down to minimum temperature over one hour), light intensity reaching full capacity of 500 μmol m^−2^ s^−1^ over 1 h and dimming to complete darkness over 1 h to mimic sunrise and sunset, respectively. At 11 days before anthesis, leaves were sampled at five time points for 24 h starting at programmed sunset: 1, 3, 6, 12, and 24 h following stress induction at sunset. To induce heat stress, from half an hour before sunset, the temperature was ramped up and reached the night-time stress temperature of 27 °C by sunset; sampling started 1 h after stress induction. The same time points were used for control plants without any stress. For each time point, all leaflets of the third trifoliate were sampled from three separate plants with a total of three biological replicates resulting in a total of 15 samples (plants) per temperature treatment, per genotype.

Total RNA was isolated as described for the developmental gene atlas. An Illumina TruSeq Stranded mRNA Library Preparation Kit was used to generate libraries. Libraries were sequenced on an Illumina HiSeq 4000 with a yield of ~35 M 50 nt reads per sample. Raw read quality was assessed using FastQC (v 0.11.5; https://www.bioinformatics.babraham.ac.uk/projects/fastqc/) and adapters and low-quality bases removed using Cutadapt^77^ (v 1.18) using the following parameters:-q 20 -n 2 –trim-n –minimum-length 31. Frijol Bayo RNA-seq reads were mapped to the Frijol Bayo *P. acutifolius* reference genome and Amadeus-77 RNA-seq reads were mapped to G19833 (Chaucha chuga) *P. vulgaris* V2.1 genome^18^ using HISAT2^89^ (v 2.1.0). HTseq^91^ (v 0.9.1) was used to obtain the read counts for each gene based on a gtf file for each genotype. Expression abundances (FPKM) were quantified using Cufflinks^90^ (v 2.2.1). Pairwise Pearson’s correlation was calculated for all samples using the “cor” function in R. *P*-values were calculated using the R package “psych” (Supplementary Figs. 16 and 17). Samples with a Pearson Correlation Coefficient of less than 90% were removed from differential gene expression; this resulted in the removal of one sample from the tepary RNA-seq libraries (Control_3h_B2) and four samples from the common bean RNA-seq libraries (Control_3h_B2, Control_12h_B3, Stress_3h_B1, Stress_12h_B1). Read counts were used for differential gene expression analysis for each species. Genes with count per million (cpm) ≥1 in at least three samples were retained. Differential gene expression analysis was conducted in R using the Limma package with voom transformation^92–94^. Multiple contrasts were considered consisting of differences between the stress and control conditions at each time point. The parameters used for decideTests to get the final table of differentially expressed genes were as follows: method = “global”, adjust.method = “BH”, p.value=0.05, lfc = 1. Hierarchical clustering was applied to separate the upregulated and downregulated genes at each time point. For each cluster, the topGO package (v2.36.0; https://bioconductor.org/packages/release/bioc/html/topGO.html) was used for GO enrichment using the classic method Fisher’s exact tests to assign a *P-value* to the GO term class; GO terms with a *P*-value < 0.05 were considered significant. To illustrate the expression networks, gene expression values generated from the Limma voom transformation were fed into ARACNeAP (version 20190423)^95^. All differentially expressed transcription factors within each time point were also used as input for ARACNe. A threshold for Mutual Information was calculated using *P*-value of 0.01 and seed of 1. ARACNe was run on 100 bootstraps of the input matrix with *P-value* of 0.01 and then consolidated into a single final network file. The network was imported into Cytoscape 3.7.1^96^ to create the network images.

To determine whether prolonged and elevated temperatures during flowering would invoke the heat shock response, Frijol Bayo and Adamdeus-77 were grown under controlled growth chamber conditions with a 12 h photoperiod, 60% relative humidity, 29 °C day/20 °C night temperature until 11 days prior to anthesis at which they were shifted to a 36 °C day/32 °C night temperature regime. At flowering, leaflets of 7th to 8th trifoliate, which either completely developed or developed under heat stress subsequent to its emergence, were sampled for RNA. Total RNA was isolated as described for the developmental gene atlas. Illumina TruSeq Stranded mRNA libraries were generated and sequenced on an Illumina HiSeq 4000 in single-end mode (50 nt reads). Raw reads were assessed for quality using FastQC (v0.11.8; https://www.bioinformatics.babraham.ac.uk/projects/fastqc/) and adapters and low-quality bases were removed using Cutadapt^77^ (v2.8) with the following parameters: -q 30,30 -m 30 –trim-n -n 2. Cleaned RNA-seq reads were aligned using HISAT2 (v2.1.0)^89^ to their cognate reference genomes. Expression abundances (FPKM) were generated using Cufflinks^90^ (v2.2.1) and read counts per gene were calculated using HTseq^91^ (v0.11.2). Read counts were then used for differential gene expression analysis using the DESeq2 Package^97^ (v1.22.2) within R (v3.5.0). Within DESeq2, the contrasts function was used to assess whether log2 fold-change was equal to 0 for pairs of contrasts. A strict log2 fold-change threshold of 2, along with an adjusted *P*-value cutoff of 0.01 was used for extracting differentially expressed genes. Furthermore, the “lfcshrink” function within DESeq2 was used to help restrain the high log2 fold changes of genes that had low expression values.

### Evolutionary analyses

Orthologous pairs between *P. vulgaris* G19833, and cultivated (Frijol Bayo) and wild (W6 15578) *P. acutifolius* were identified by reciprocal best BLASTP hits (e-value cutoff = 10^−10^). MCScanX^98^ (v2) with default options was used to scan for collinear blocks between the assemblies. The collinear blocks were visualized using SynVisio (https://synvisio.github.io/). To determine the orthologous and paralogous groups in the Phaseoleae tribe, OrthoFinder2^99^ (v2.2.7) was run with default search parameters and the representative proteins from *A. thaliana* (TAIR10)^85^, *Medicago truncatula* (Mt4.0v1)^100^, *Cajanus cajan* (v1)^101^, *Glycine max* (Wm82.a2.v1)^102^, *Vigna unguiculata* (v1.1)^103^, *P. vulgaris* (v2.1), *P. acutifolius* Frijol Bayo, and *P. acutifolius* W6 15578. The R package ‘UpsetR’ (v1.4.0)^104^ was used to plot the results. An ultrametric tree was constructed using the OrthoFinder rooted species tree with the ‘make_ultrametric.py’ python script in OrthoFinder. The root age was estimated to be 120 million years ago based on data from two separate studies^105,106^. CAFE^107^ (v4.2.1; –filter -p 0.01 -r 10000) was used with the ultrametric tree and the identified gene families from OrthoFinder to characterize the expanded and contracted gene families. The Bioconductor R package ‘topGO’ (v2.36.0; https://bioconductor.org/packages/release/bioc/html/topGO.html) was used to perform a Fisher’s exact test with the classic method for gene ontology (GO) enrichment analyses. The p-values were adjusted using the false discovery rate (FDR) correction and GO terms with adjusted *P*-values < 0.05 were retained.

Protein sequences of *P. acutifolius* (Frijol Bayo and W6 15578) were aligned (BLASTP^108^; with E-value cutoff of 1E-10) with protein sequences of *P. vulgaris* G19833 (Phytozome; Pvulgaris_442_v2.1.protein.fa), *G. max* (EnsemblPlants; Glycine_max_v2.1.pep.all.fa) and *V. unguiculata* (Phytozome; Vunguiculata_469_v1.1.protein.fa). Putative orthologs between different species were identified by using the reciprocal best BLAST hit method. Estimation of the level of synonymous substitution (*Ks*) between orthologous pairs was performed using pairwise_*kaks*.PLS (v 1.8; https://github.com/cybersiddhu/bioperl-live/blob/master/scripts/utilities/pairwise_kaks.PLS). This script takes an input of cDNA sequences, verifies that they do not contain stop codons, aligns them in protein space, projects the alignment back into cDNA, and estimates the Ka (non-synonymous) and Ks (synonymous) substitutions based on the maximum likelihood method of Yang with the PAML package^109^.

*Ks* values less than 0.001 are affected by rounding errors that result in spurious peaks. So we used *Ks* values greater than 0.001 for generating histograms. Gaussian mixture models were fitted by the R package Mclust (v5.4.6) and the number of Gaussian components (G), the mean of each component, and fractions of data were recoded. The best fitting model to the data was determined and tested using the Bayesian Information Criterion and *χ*^2^ tests, respectively. In the case of skewed peaks resulting from overlapping Gaussian components, we combined and represented the overlapping components with the mean and standard deviation of the Gaussian mixtures.

Based on the widely reported divergence time between soybean and common bean of ~19.2 Mya, the global mutation rate for leguminous species (*λ*) was calculated to be 8.3 × 10^−9^, using the formula *λ* = *Ks*/2*T*, where *T* is the age of divergence (19.2 Mya) and *Ks* is the geometric mean of synonymous distances between orthologous gene pairs of soybean and common bean (*Ks* = 0.32). Using this mutation rate (*λ* = 8.3 × 10^−9^) and the geometric mean *Ks* of peaks, the age of divergence between other leguminous species was estimated as *T* = *Ks*/2λ.

### Disease resistance gene analyses

The domain structure of the predicated primary proteins of *P*. *acutifolius* Frijol Bayo, *P. acutifolius* W6 15578, and *P*. *vulgaris* (Phytozome v2.1) was analyzed with the *HMMERSEARCH* software using the PfamA domain database. All proteins from *P*. *acutifolius* Frijol Bayo and *P*. *vulgaris* containing an NB-ARC domain with an E-value <1.00E−60 were identified. The NB-ARC domains from the three *P. acutifolius* cultivated and four *P. vulgaris* proteins that met the criteria were used as seed sequences to create a *Phaseolus* NB-ARC domain profile using the hmmbuild program in HMMER (v3.2.1)^84^. The proteins from the species were again scanned using *HMMERSEARCH* with the *Phaseolus* NB-ARC profile, and that domain output was substituted for the NB-ARC data from the original *HMMERSEARCH* search. The domain features associated with all *Phaseolus* NB-ARC-containing proteins were collated. Those domains most often associated with NLR proteins were: TIR (Pfam01582), TIR_2 (Pfam13676), LRR_3 (Pfam07725), LRR_4 (Pfam12799), LRR_5 (Pfam13306), LRR_8 (Pfam13855), and Rpw8 (Pfam05659). Proteins containing a coiled-coil (CC) domain were identified using the COILS software(v2.2)^110^ (probability ≥ 0.90). Proteins with the following core domains were discovered: CC-NB-ARC-LRR (CNL), TIR-NB-ARC-LRR (TNL), RPW8-NB-ARC-LRR (RNL), NB-ARC-LRR (NL), CC-NB-ARC (CN), TIR-NB-ARC (TN), RPW8-NB-ARC (RN), and NB-ARC NB-ARC (N). A subset of proteins containing at least one subdomain-specific amino acid signature of the following NB-ARC domain amino acid signatures of the three NB-ARC subdomains was identified: P-loop (GTTK), kinase 2 (VLDD), ARC1 (GLPL), and ARC2 (MHD). NB-ARC gene clusters were defined as containing a minimum of three NB-ARC domain-containing genes that were located <200 kb from another NB-ARC gene and with no more than nine other gene models located between any two members of the cluster. *P. acutifolius* and *P. vulgaris* clusters were considered related if a pair of gene models for the two species were reciprocal best hits based on a BLASTP analysis. Related clusters were assigned the same name. The *P. acutifolius* and *P. vulgaris* NB-ARC sequence were used for all phylogenetic analyses with the MEGA 7.0 phylogenetic analysis package^111^ (www.megasoftware.net). The NB-ARC domain amino acids sequences were aligned with the MUSCLE algorithm (v3.4)^112^. Any sequence which did not have a pairwise alignment with another sequence was removed. A neighbor-joining tree was constructed using the JTT model method, with a site coverage cutoff of 50% and 100 bootstraps.

The domain structures for all protein sequences containing an NB-ARC domain were collated on a protein-by-protein basis from the *HMMERSEARCH* output. Those protein sequences found to contain Pfam domains (*E*-value < 1.0) other than the canonical NLR sequences (TIR, TIR_2, RPW8, NB-ARC, LRR_3, LRR_4, LRR_5, and LRR_8) were identified. Those protein sequences found to contain a COILS domain were also identified. Any other Pfam domain was considered a non-canonical domain. Any non-canonical domain not overlapping with another domain was identified as an integrated domain.

### Transcription factor annotation

The protein sequences derived from the gene models of *P. vulgaris* G19833 and *P. acutifolius* (Frijol Bayo and W6 15578) were submitted to the Plant Transcription Factor Database v.4.0^113^ (planttfdb.cbi.pku.edu.cn/prediction.php) to identify the transcription factors (TF) in each of the genomes. The rules to assign a protein to a TF can be found at this WWW site: planttfdb.cbi.pku.edu.cn/help_famschema.php.

### Domestication and adaptation analyses

To identify regions of domestication and adaptation, 55 genotypes (16 wild and 39 landraces) were selected for deep sequencing from the Tepary Diversity Panel (TDP) that consisted of a collection of 425 landraces and wild accessions. Genotypes were selected based on population structure, genetic distance, putative gene pool, seed types, key breeding genotypes, and important accessions based on abiotic and biotic stress. Cultivated and wild types were originally discriminated based on domestication traits such as seed size and phaseolin patterns. For genetic differentiation, the TDP was genotyped using GBS sequencing^71^ that resulted in 28,750 SNPs. The SNPs were used to investigate population structure in the TDP, and eight subpopulations were identified. These subpopulations were assigned a name based on their geographical origin and subspecies composition (Sonora tepary, Sonora acutifolius, etc). Fifty-five genotypes that best represent the diversity among these eight subpopulations (Supplementary Data 17) were then subjected to STRUCTURE (v2.3.4) analysis because the wild types were a mixture of genotypes representing the *acutifolius* and *tenuifolius* subspecies. Further, for the domestication and adaptation analysis, it was important to ensure the genotypes of these two subspecies were not significantly different at the genome level. Based on this analysis, the optimum number of subpopulations for these 55 genotypes was three which corresponded to one group of landraces and two groups of wild type. However, at a *K* value > 0.60 for each subpopulation and the corresponding genotypes, it was clear that each wild subpopulation was actually a mixture of each subspecies. Therefore, for our domestication analysis, the 55 genotypes were simply classified as either wild or domesticated.

Domestication (a reduction/loss of diversity) and adaptation (acquiring new alleles or allele frequency change from the neutral genome) analyses were performed on SNPs called from the alignments of whole-genome resequencing data of each genotype (average coverage of 20×) against the tepary Frijol Bayo landrace reference genome. The diversity parameters were calculated over 100k–10 kb sliding windows. π (the average pairwise nucleotide differences in a sample), Ɵ (number of segregating sites in a sample), and unique polymorphisms in wild accessions were calculated to identify genomic regions associated with domestication. The distribution of the SNPs in which one of the alleles was present in the wild subpopulation and a different allele was present in the landrace subpopulation was calculated to detect the adaptation genomic regions. The genomic regions that were significantly different were then investigated to identify candidate genes (using the gene models from the Frijol Bayo reference genome). The predicted effect of polymorphism on a gene was determined using snpEff (v4.0)^114^, and the associated gene ontology for a gene was determined using topGO (v2.38.1) (https://bioconductor.org/packages/release/bioc/html/topGO.html).

### Reporting summary

Further information on research design is available in the Nature Research Reporting Summary linked to this article.

## Supplementary information

Supplementary Information

Reporting Summary

Description of Additional Supplementary Files

Supplementary Data 1

Supplementary Data 2

Supplementary Data 3

Supplementary Data 4

Supplementary Data 5

Supplementary Data 6

Supplementary Data 7

Supplementary Data 8

Supplementary Data 9

Supplementary Data 10

Supplementary Data 11

Supplementary Data 12

Supplementary Data 13

Supplementary Data 14

Supplementary Data 15

Supplementary Data 16

Supplementary Data 17

Supplementary Data 18

Supplementary Data 19

## Data Availability

Data supporting the findings of this work are available within the paper and the Supplementary Information files. A reporting summary for this article is available as a Supplementary Information file. The data sets generated and analyzed during this study are available from the corresponding author upon request. Raw sequence reads are available in the National Center for Biotechnology Information Sequence Read Archive under BioProject ID PRJNA607288. Large associated data sets include (i) genome assembly, gene annotation (genes, gene models, transcripts, peptides), and associated gff files; (ii) gene expression matrices and differentially expressed genes; and (iii) orthologous groups are available in the Dryad Digital Repository [10.5061/dryad.6q573n5w2]. The genome assemblies with their annotation are also available at Phytozome [https://phytozome-next.jgi.doe.gov/info/Pacutifolius_v1_0; https://phytozome-next.jgi.doe.gov/info/PacutifoliusWLD_v2_0]. External data sets used in this study include: *G. max* proteome (EnsemblPlants; Glycine_max_v2.1.pep.all.fa; https://plants.ensembl.org/); PFAM (v32; http://pfam.xfam.org/); Phytozome genome/proteomes (*A. thaliana* proteome, TAIR10); *Cajanus cajan* (v1); *Glycine max* (Wm82.a2.v1); *Medicago truncatula* (Mt4.0v1); *P. vulgaris* V2.1; *Vigna unguiculata* (v1.1); https://phytozome.jgi.doe.gov/pz/portal.html; RepBase (v20150807) Viridiplantae repeats; Swiss-Prot (2015_08; https://www.uniprot.org/); Plant Transcription Factor Database v.4.0 (planttfdb.cbi.pku.edu.cn/prediction.php). The germplasm is available from the U.S. Department of Agriculture Germplasm Resources Information Network (https://www.ars-grin.gov/). Source data are provided with this paper.

## Source data

Source Data
