# Supplementary material for: The tepary bean genome provides insight into evolution and domestication under heat stress
Source: Nat Commun. 2021 May 11;12:2638. doi: 10.1038/s41467-021-22858-x (PMC8113540; doi:10.1038/s41467-021-22858-x)
Supplement: Supplementary file 1 — Supplementary Information [file 41467_2021_22858_MOESM1_ESM.pdf]

The tepary bean genome provides insight into evolution and domestication under heat stress

Moghaddam *et al.*

Supplementary Table 1. SNP diversity among pooled sequencing populations.

**Type of SNP**	**# of SNP**	**% of SNP**		**% of SNPs in the genes**
Unique wild SNPs	2,247,877	66.56	SNPs polymorphic in wild and monomorphic in landraces; domestication SNPs	21
Unique landrace SNPs	94,316	2.79	SNPs polymorphic in landrace and monomorphic in wild	
Shared SNPs	1,020,744	30.22	SNPs polymorphic in both wild and landraces	
Fixed SNPs	14,502	0.43	Alternate SNPs alleles fixed in wild and landrace subpopulations; adaptation SNPs	

Supplementary Table 2. Genomic libraries included in the *Phaseolus acutifolius* Frijol Bayo genome assembly and their respective assembled sequence coverage levels in the final release.

*Average read length of PACBIO reads.	Sequencing Platform	Assembly	Average Read/Insert Size	Read Number	Assembled Sequence Coverage (x)
IKFX	Illumina-X10	1	480	540,535,272	159.2
IPHM	Illumina-HIC	1	N/A	415,119,662	122.3
	PACBIO	1	6,044*	6,524,706	101.28

Construction of the scaffold assembly. A total of 6,524,706 PACBIO reads (101.28x) were assembled using MECAT (Xiao et al., 2017), and formed the starting point of the version 1.0 release. The 415,119,662 Illumina HIC reads were used to identify misjoins and verify order/orientation of the final chromosomes. The 540,535,272 Illumina sequence reads (159.2x sequence coverage) were used for fixing homozygous snp/indel errors in the consensus.

Supplementary Table 3. PACBIO library statistics for the libraries included in the *Phaseolus acutifolius* Frijol Bayo genome assembly and their respective assembled sequence coverage levels.

Cutoff	Number of Reads	Basepairs	Average Read Length	Coverage
0	6,524,706	55,704,493,133	6,044	101.28x
1,000	5,580,620	55,244,311,435	7,602	100.44x
2,000	4,946,870	54,309,559,757	8,780	98.74x
3,000	4,436,889	53,044,185,483	9,813	96.44x
4,000	4,004,050	51,534,238,865	10,776	93.70x
5,000	3,621,188	49,815,015,794	11,720	90.57x
6,000	3,276,590	47,922,956,043	12,661	87.13x
7,000	2,964,697	45,898,492,295	13,606	83.45x
8,000	2,679,456	43,761,245,612	14,552	79.57x
9,000	2,418,001	41,540,772,704	15,509	75.53x
10,000	2,175,218	39,236,453,480	16,438	71.34x
11,000	1,954,900	36,925,351,607	17,271	67.14x
12,000	1,757,727	34,659,838,277	18,027	63.02x
13,000	1,580,681	32,448,533,666	18,755	59.00x
14,000	1,421,032	30,294,554,782	19,479	55.08x
15,000	1,277,076	28,208,477,356	20,214	51.29x
16,000	1,144,929	26,160,814,771	20,962	47.57x
17,000	1,013,690	23,995,326,259	21,808	43.63x
18,000	882,392	21,698,158,193	22,763	39.45x
19,000	762,315	19,478,099,047	23,759	35.41x

Supplementary Table 4. Summary statistics of the initial output of the *Phaseolus acutifolius* Frijol Bayo ARROW polished MECAT assembly. The table shows total contigs and total assembled basepairs for each set of scaffolds greater than the size listed in the left hand column.

Minimum Scaffold Length	Number of Scaffolds	Number of Contigs	Scaffold Size	Basepairs	% Non-gap Basepairs
5 Mb	34	34	311,951,900	311,951,900	100.00%
2.5 Mb	58	58	392,857,741	392,857,741	100.00%
1 Mb	91	91	444,773,400	444,773,400	100.00%
500 Kb	118	118	464,167,813	464,167,813	100.00%
250 Kb	142	142	472,532,661	472,532,661	100.00%
100 Kb	250	250	488,377,528	488,377,528	100.00%
50 Kb	474	474	503,554,767	503,554,767	100.00%
25 Kb	925	925	519,757,695	519,757,695	100.00%
10 Kb	1,092	1,092	523,127,949	523,127,949	100.00%
5 Kb	1,092	1,092	523,127,949	523,127,949	100.00%
2.5 Kb	1,092	1,092	523,127,949	523,127,949	100.00%
1 Kb	1,092	1,092	523,127,949	523,127,949	100.00%
0 bp	1,092	1,092	523,127,949	523,127,949	100.00%

Supplementary Table 5. Final summary assembly statistics for the *Phaseolus acutifolius* Frijol Bayo chromosome scale assembly.

Scaffold total	155
Contig total	667
Scaffold sequence total	512.6 Mb (1.0% gap)
Chromosome Sequence	502.4 Mb
Contig sequence total	507.5 Mb
Scaffold N/L50	5 / 45.2 Mb
Contig N/L50	25 / 6.2 Mb

Supplementary Table 6. Wild tepary bean (W6 15578) sequencing libraries.

Library type	Insert size	Read length(bp)	Depth (X)
PCR-free PE library (PE250X2)	450-470bp	2 x 250	150
PCR-free PE library (PE150X2)	700–800bp	2 x 150	38
MP (Nextera™MP Gel Plus)	2-4kbp	2 x 150	50
MP (Nextera™MP Gel Plus)	5-7kbp	2 x 150	47
MP (Nextera™MP Gel Plus)	8-10kbp	2 x 150	48
10X genomics™Chromium™	N/A	2 x 150	175
Total			508

Supplementary Table 7. Final summary assembly statistics for the *Phaseolus acutifolius* (W6 15578) chromosome scale assembly.	
Scaffold total	609
Contig total	29,934
Scaffold sequence total	662 Mb (1.6% gap)
Chromosome Sequence	466.8 Mb
Contig sequence total	651.1 Mb
Scaffold N/L50	8 / 42.6 Mb
Contig N/L50	3492 / 47 Kb

a) Tepary Bean – cultivated accession Frijol Bayo (684Mb)

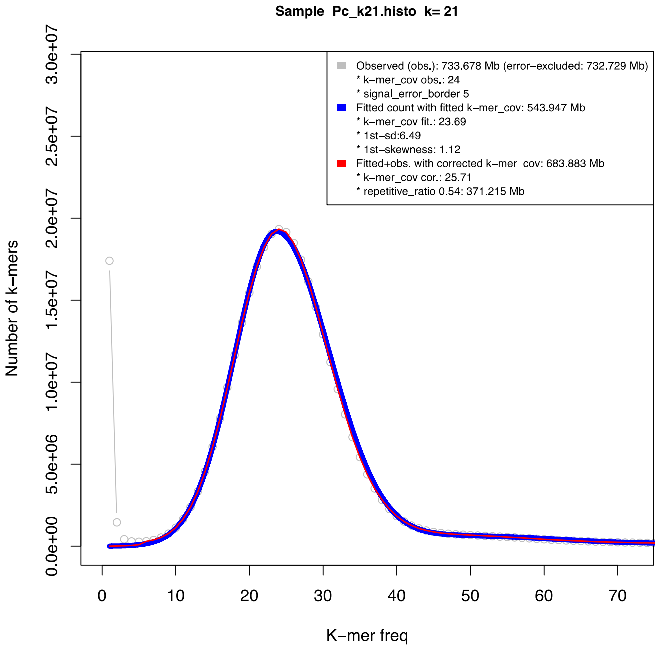

b) Tepary Bean – wild accession W6 15578 (651Mb)

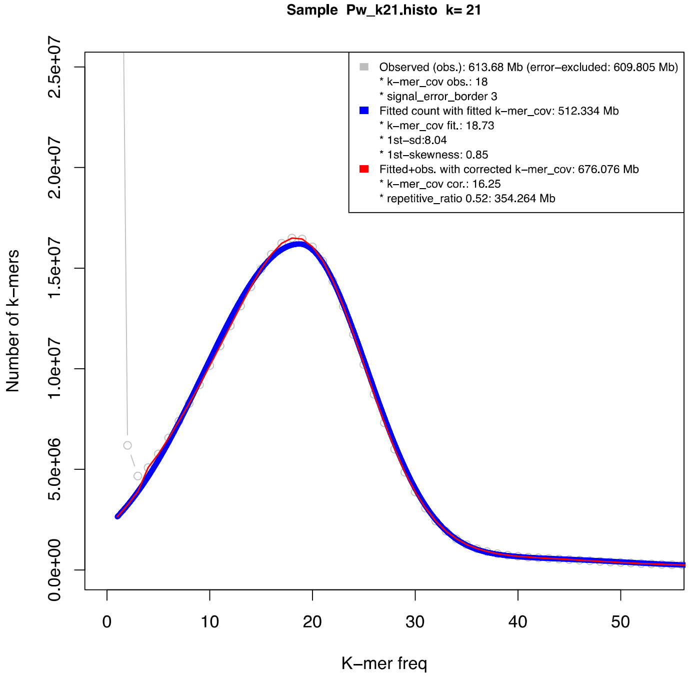

Supplementary Figure 1. Estimated genome size of wild and cultivated *P. acutifolius* genomes using k-mer distributions.

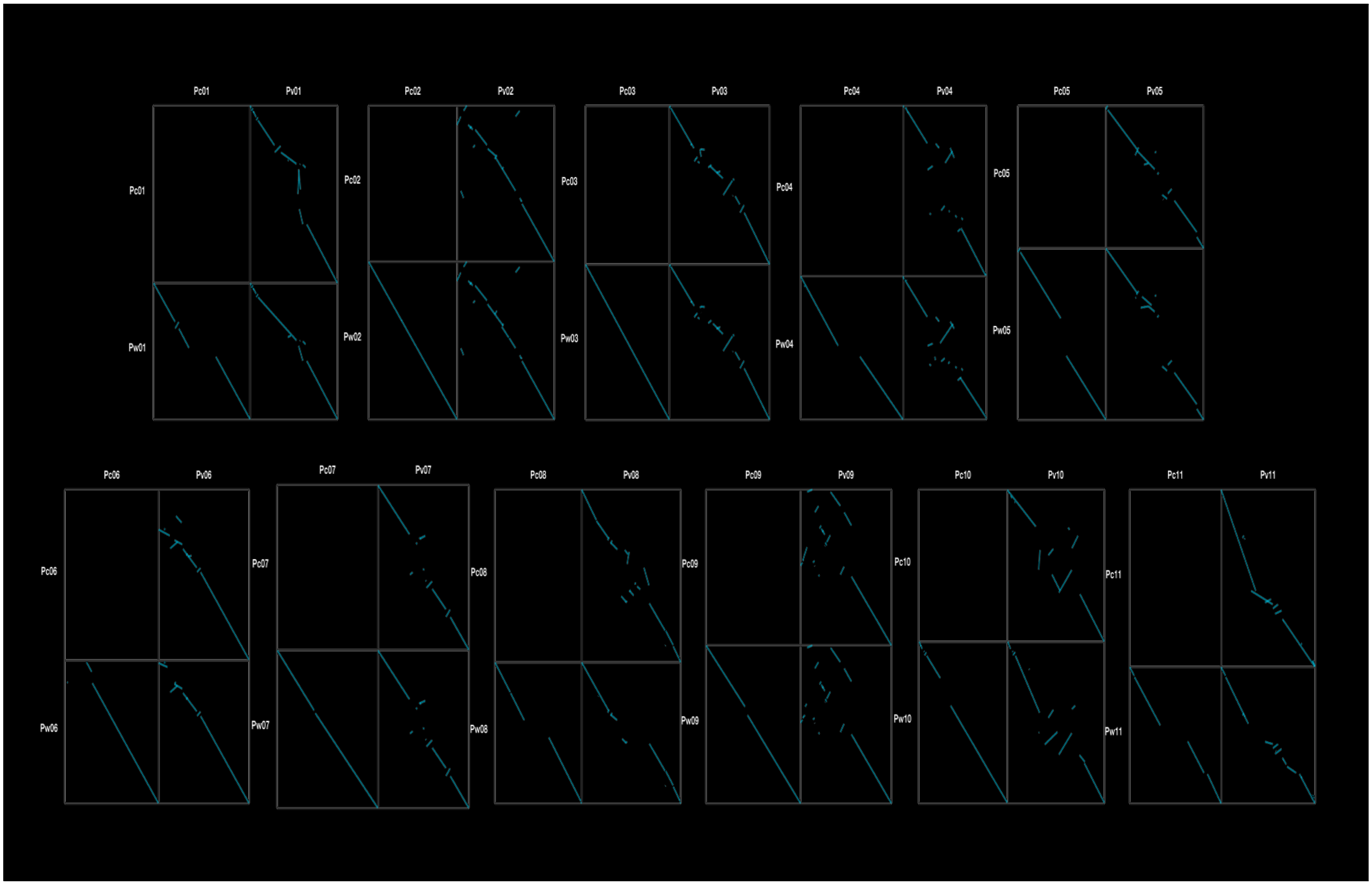

Supplementary Figure 2. Synteny between chromosomes of *Phaseolus vulgaris* G19833 (Pv) and *Phaseolus acutifolus* (cultivated (Frijol Bayo): Pc; wild (W6 15578): Pw).

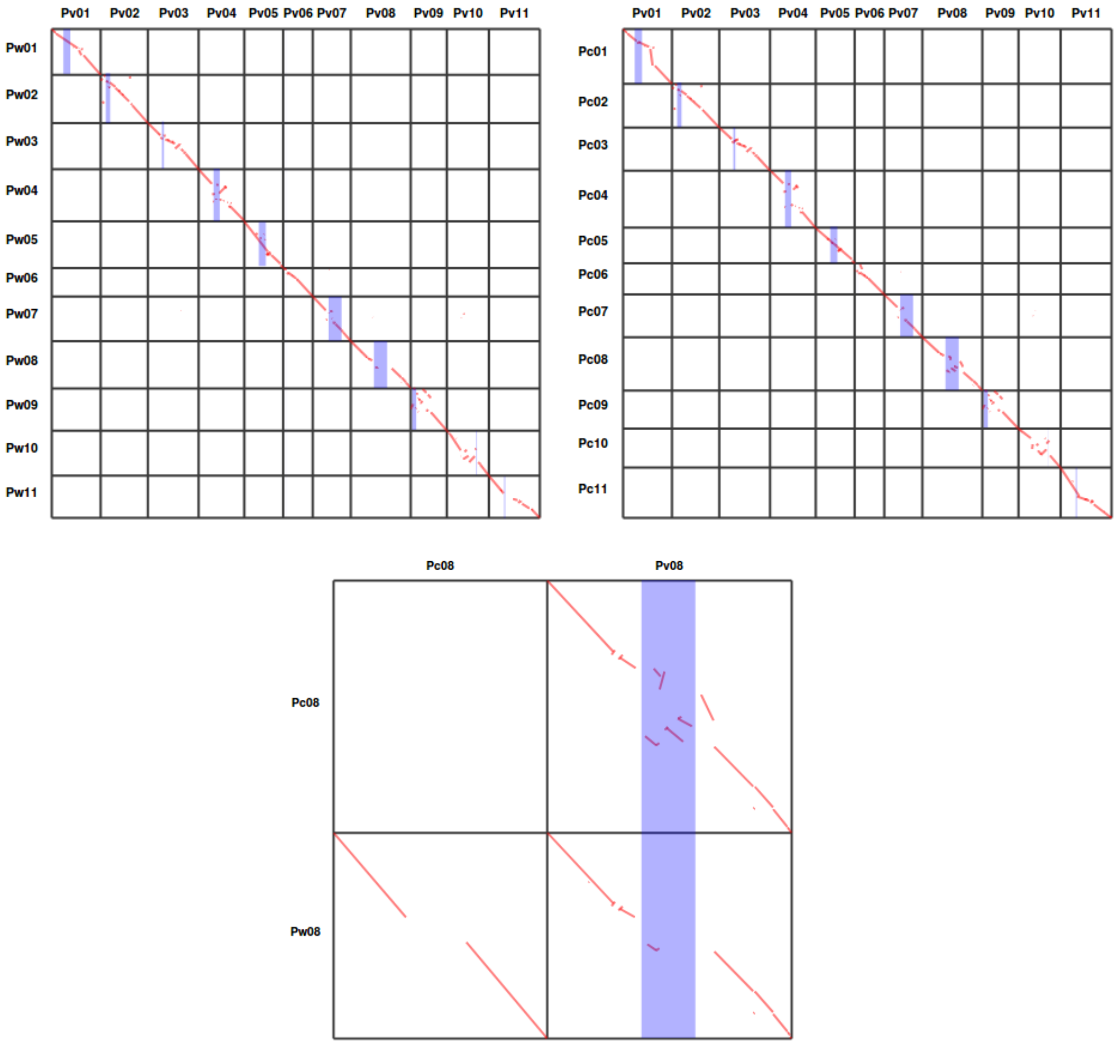

Supplementary Figure 3. Comparisons of wild (Pw; top, left) and cultivated (Pc; top, right) tepary bean chromosomes with *Phaseolus vulgaris* chromosomes (Pv). Chromosome 8 is highlighted in the lower image. Purple shaded boxes delineate centromeric regions in *P. vulgaris* as annotated in Schmutz *et al.* [3].

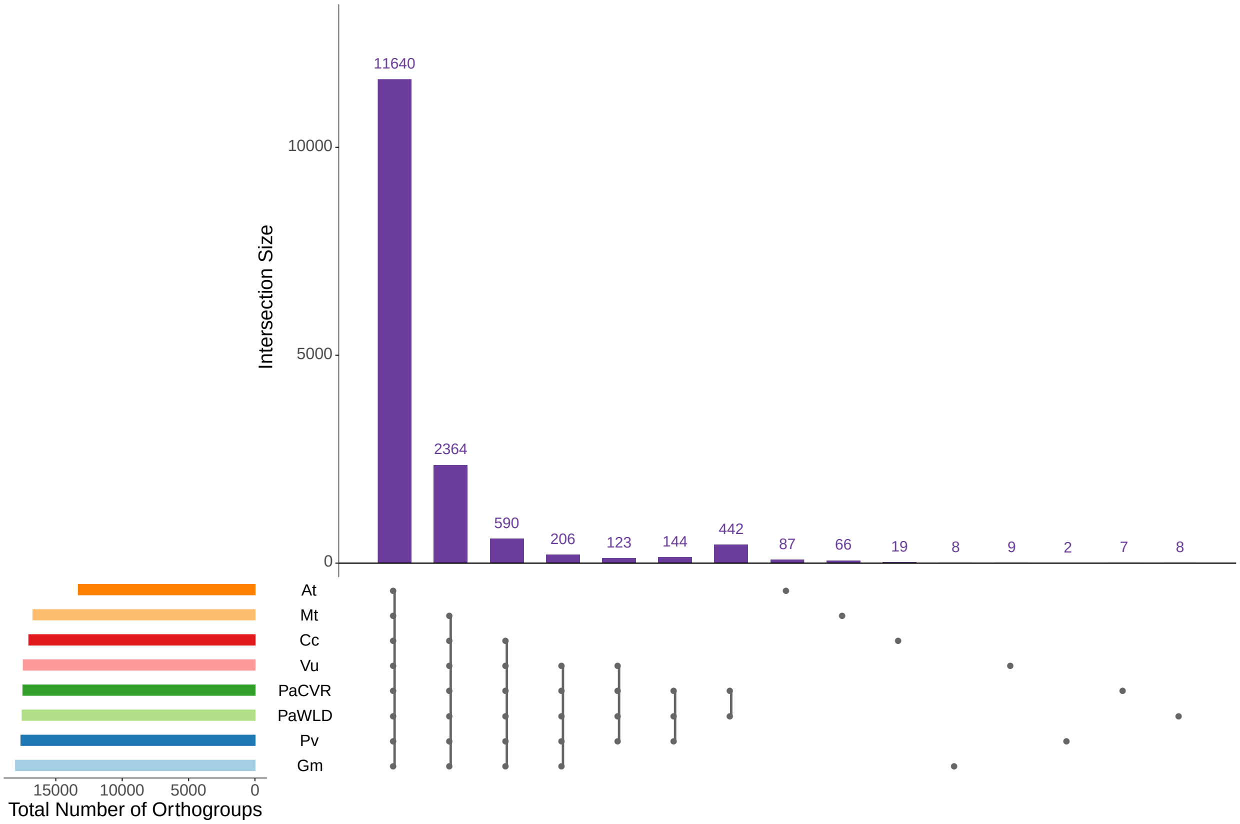

Supplementary Figure 4. Orthologous and paralogous groups shared among species families. The number of orthologous and paralogous groups identified via OrthoFinder are plotted in the main bar graph with the species present in the groups indicated below the graph. 'AT' refers to *Arabidopsis thaliana*, 'Mt' refers to *Medicago truncatula*, 'Cc' refers to *Cajanus cajan*, 'Vu' refers to *Vigna unguiculata*, 'PaCVR' refers to cultivated *Phaseolus acutifolius* Frijol Bayo, 'PaWLD' refers to wild *P. acutifolius* W6 15578, 'Pv' refers to *P. vulgaris* G19833, and 'Gm' refers to *Glycine max*. The groups are separated by their evolutionary classification with the left most bar representing groups present in Rosids and the right most bar containing at least two accessions representing the single species *P. acutifolius*. Numbers above each bar indicate the number of orthologous and paralogous groups present in that classification. The secondary bar graph on the left indicates the total number of orthologous and paralogous groups present in each species. Source data underlying Supplementary Figure 4 are provided in the Dryad Digital Repository associated with this publication (https://doi.org/10.5061/dryad.6q573n5w2).

a)

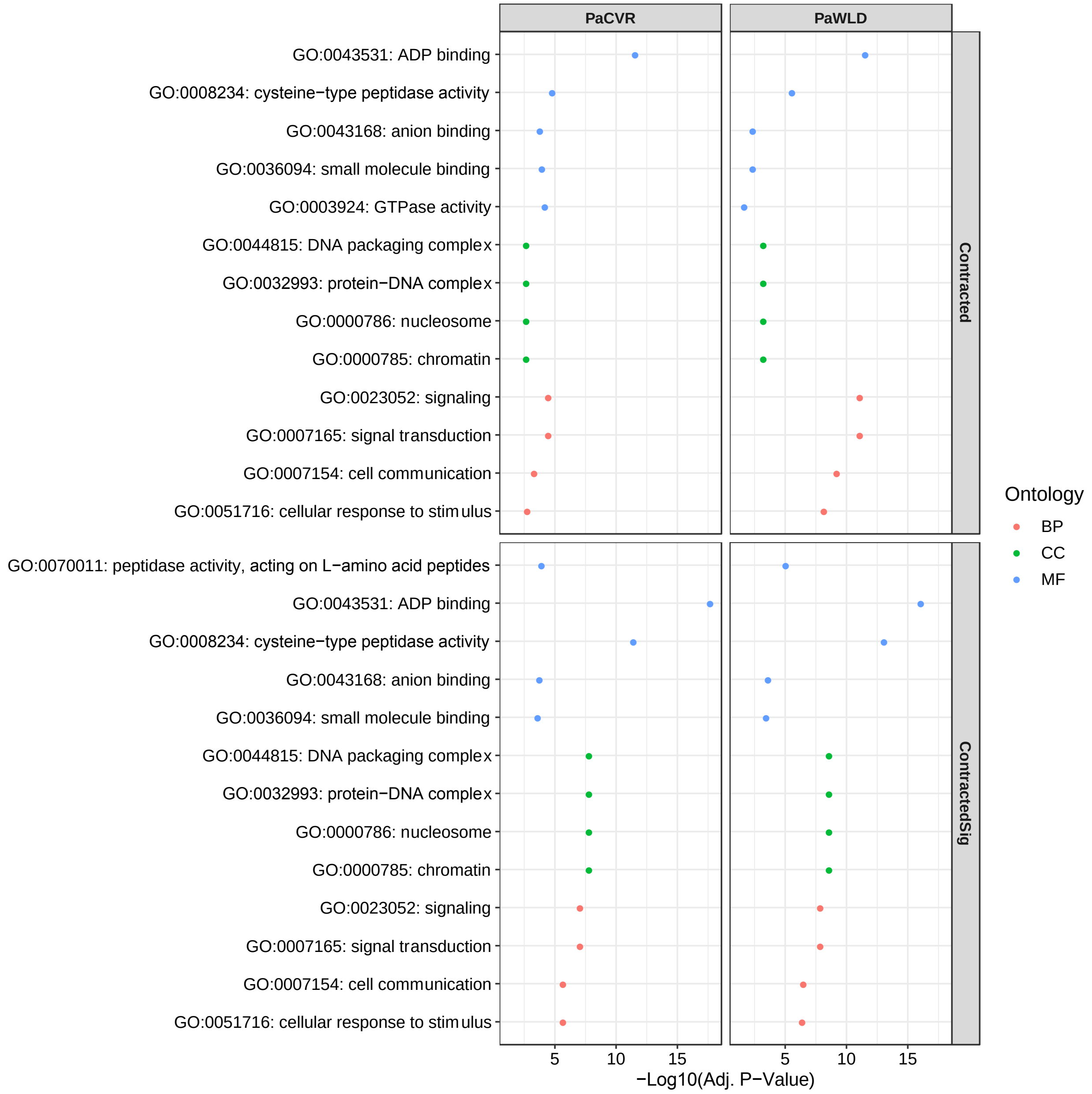
PaCVR
PaWLD
Contracted
ContractedSig
GO:0043531: ADP binding
GO:0008234: cysteine−type peptidase activity
GO:0043168: anion binding
GO:0036094: small molecule binding
GO:0003924: GTPase activity
GO:0044815: DNA packaging complex
GO:0032993: protein−DNA complex
GO:0000786: nucleosome
GO:0000785: chromatin
GO:0023052: signaling
GO:0007165: signal transduction
GO:0007154: cell communication
GO:0051716: cellular response to stimulus
GO:0070011: peptidase activity, acting on L−amino acid peptides
5
10
15
−Log10(Adj. P−Value)
Ontology
BP
CC
MF

b)

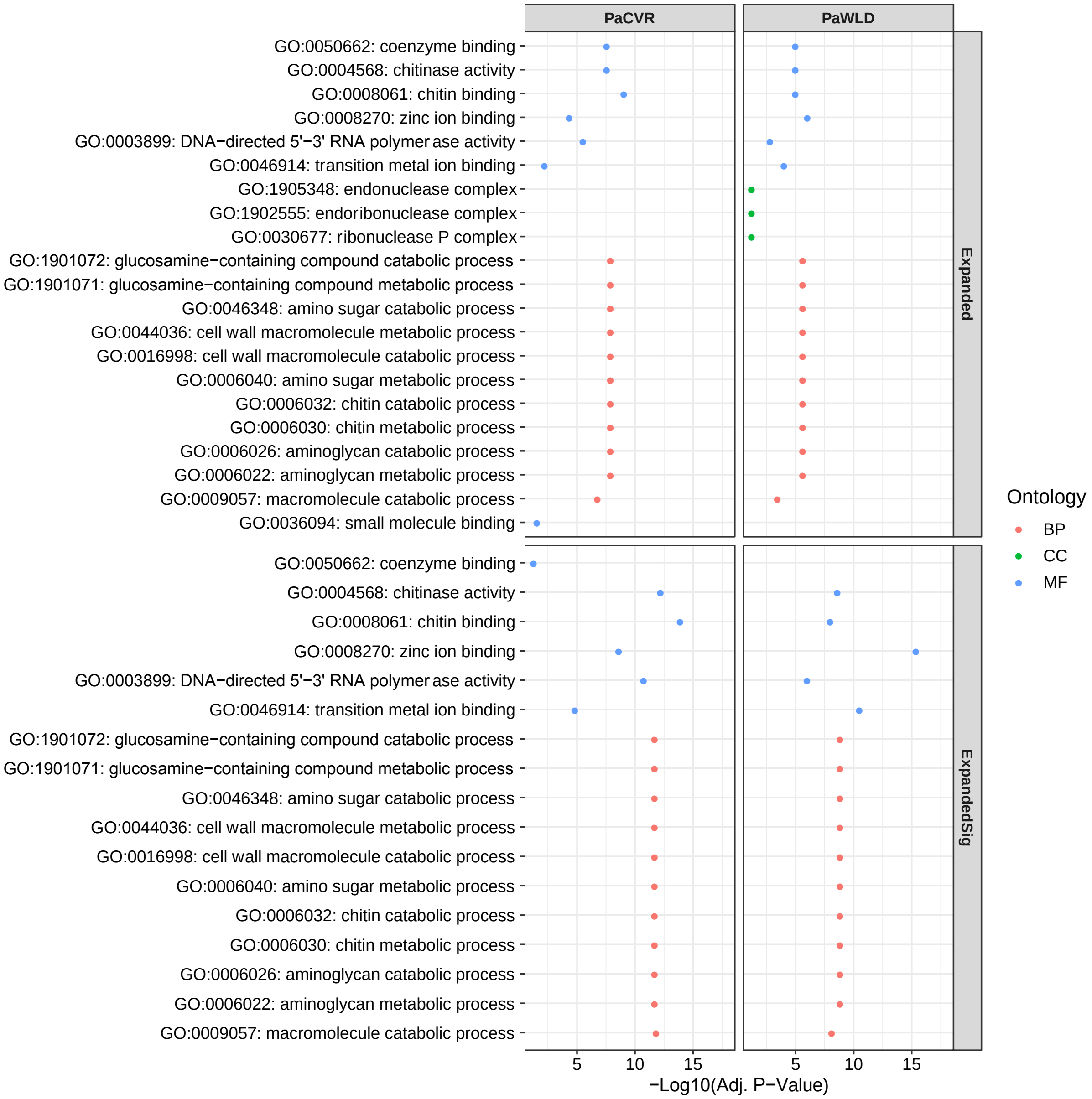

Supplementary Figure 5. Gene ontology (GO) enrichment for the expanded and contracted gene families in tepary compared to common bean. Gene Ontology enrichment analysis for the genes in expanded, contracted, significantly expanded, and significantly contracted gene families in tepary compared to common bean. The classic Fisher's exact tests (one-sided) was used for analysis. False Discovery Rate (FDR) correction was used to adjust the *P*-values and GO terms with adjusted *P*-values < 0.05 were retained. The top three significant GO terms for each family type and ontology category are on the y-axis and the significance of each term (-log$_{10}$(Adj. P-value)) is on the x-axis. The points are colored according to their ontology category and separated both by the tepary accession and by the gene family type. BP: Biological process; CC: Cellular compartment; MF: Molecular function.

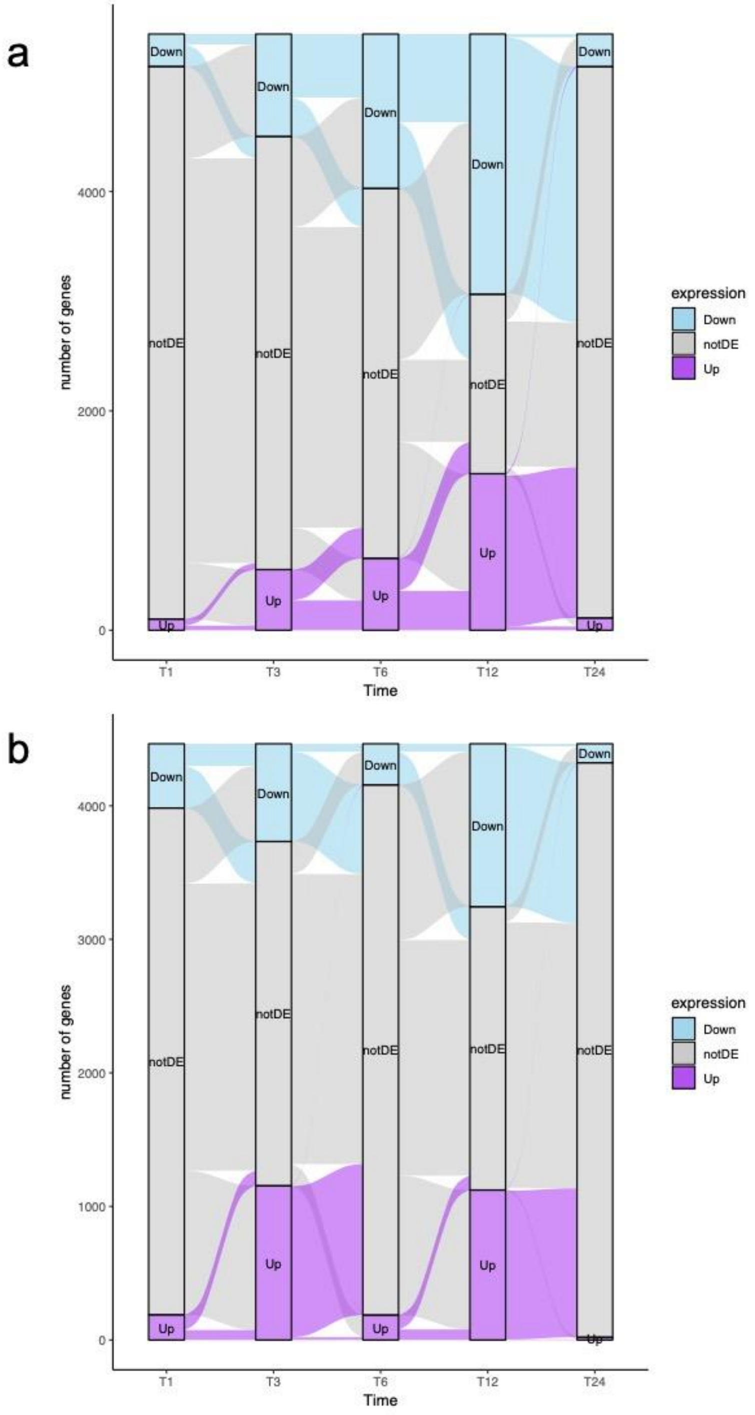

Supplementary Figure 6. Alluvial diagram of differentially expressed genes in Frijol Bayo (tepary bean) (a) and Amadeus-77 (common bean) (b) in a 24-hour time course of heat stress. Genes with 2-fold difference in their relative expression are consider differentially expressed. Blue and purple: up- and down-regulated genes under heat stress relative to control conditions, respectively. Gray: no change in gene expression level. Source data underlying Supplementary Figure 6 are provided in the Dryad Digital Repository associated with this publication (https://doi.org/10.5061/dryad.6q573n5w2).

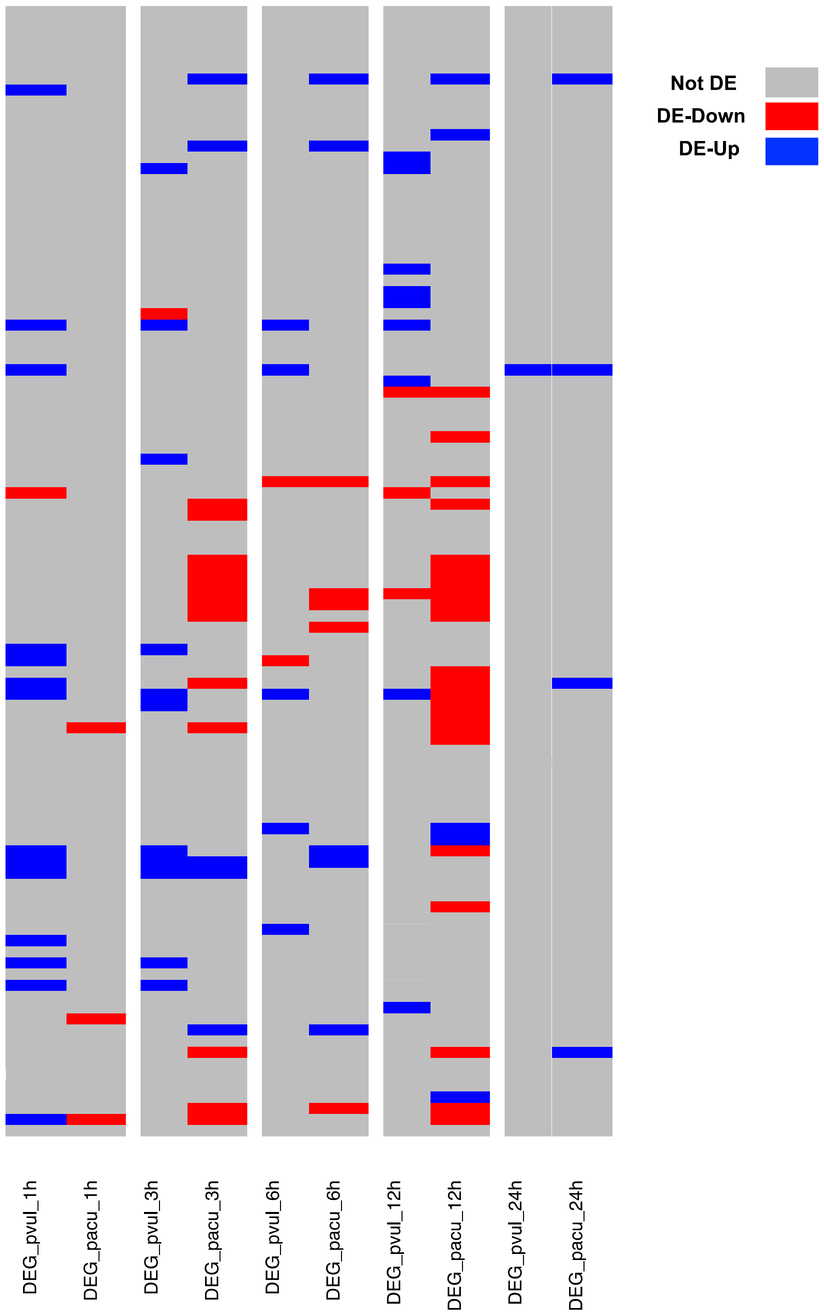

Supplementary Figure 7. Differentially expressed HSP (Heat Shock Protein) and HSF (Heat Shock Factor) Arabidopsis homologs in Frijol Bayo (tepary bean) and 'Amadeus-77' (common bean) under a 24-hour time course heat stress and control conditions. Genes with 2-fold difference in their relative expression are considered differentially expressed. Blue and red: up- and down- regulated genes under heat stress relative to control conditions, respectively. Gray: no change in gene expression level. DEG: differentially expressed genes, pvul: *Phaseolus vulgaris* (Amadeus-77), pacu: *Phaseolus acutifolius* (Frijol Bayo). Source data are provided as a Source data file.

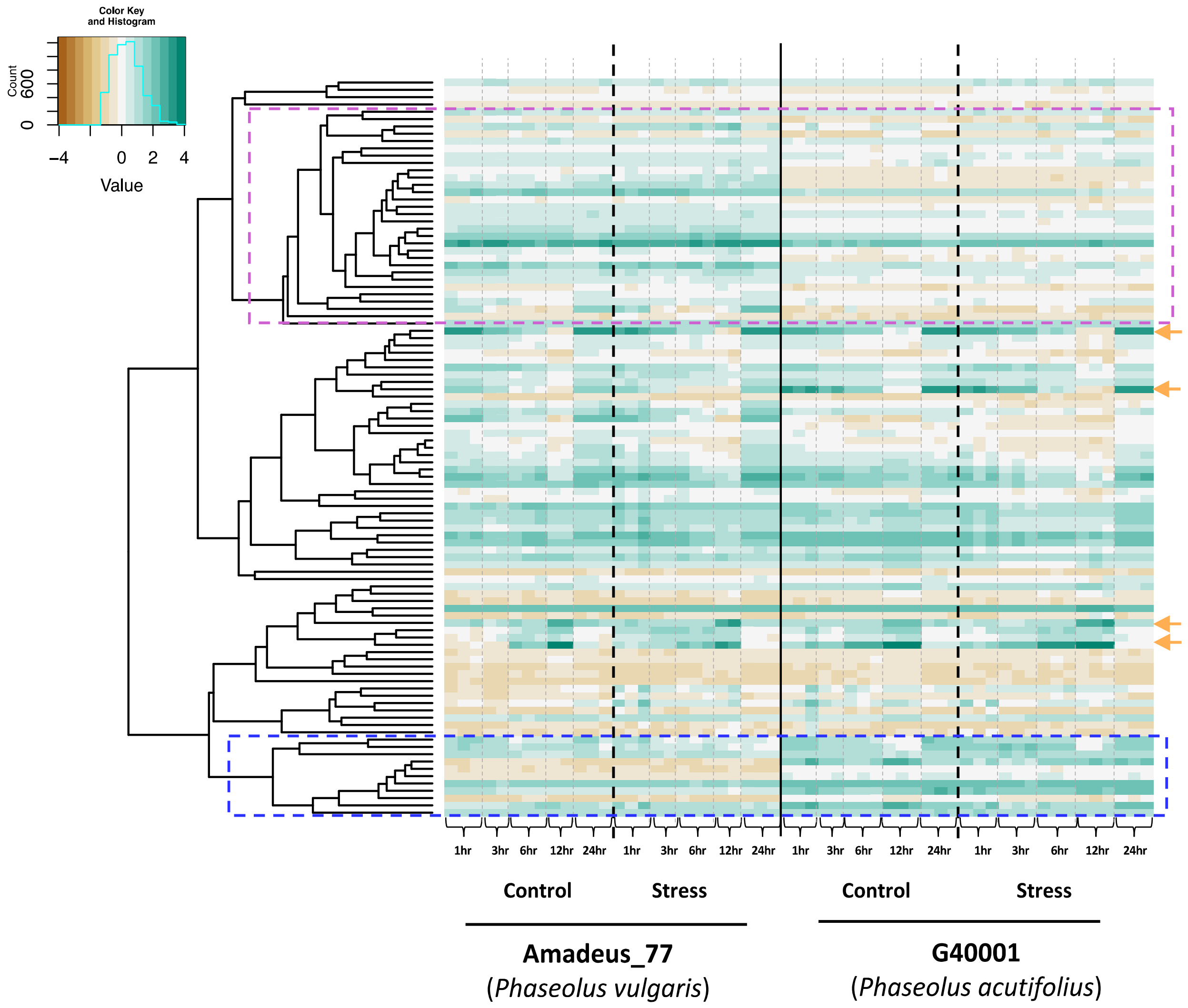

Supplementary Figure 8. Overview of the expression of HSP (Heat Shock Protein) and HSF (Heat Shock Factor) Arabidopsis homologs in Frijol Bayo (tepary bean) and ‘Amadeus-77’ (common bean) in a 24-hour time course under heat stress and control conditions. Log2 FPKM data is z-transformed across all the genes in every sample to make the across sample comparison statistically possible. The y-axis shows hierarchical clustering of all the genes based on expression values. The x-axis indicates the treatment conditions and time points in each species. Two main clusters in the dashed boxes indicate clusters of genes with different expression patterns between Frijol Bayo and ‘Amadeus-77’ regardless of the treatment condition. Gold arrows indicate the genes whose expression is affected by the time of the day across all samples (circadian rhythm). Source data are provided as a Source data file.

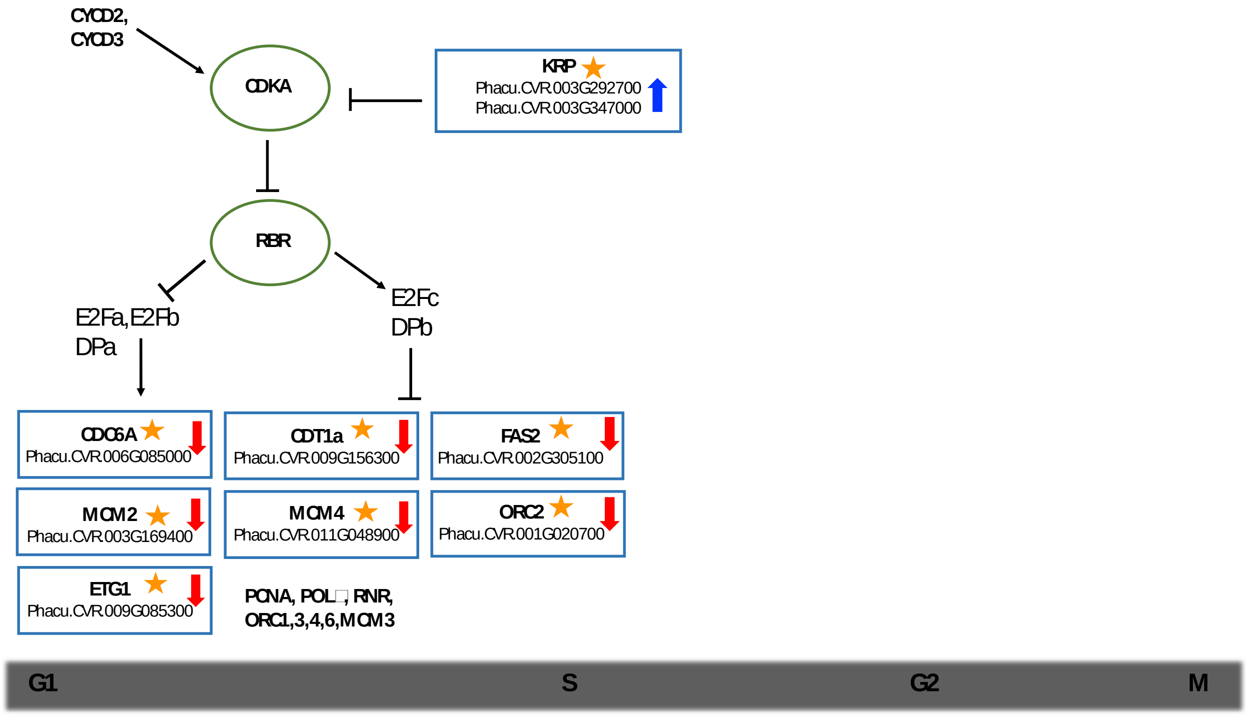

Supplementary Figure 9. Simplified pathway controlling the G1/S transition in Arabidopsis based on Gutierrez [2]. Arabidopsis KPR7 is a cyclin-dependent kinase (CDK) inhibitor, and a negative regulator of cell division which is up-regulated at 1hr after stress induction in Frijol Bayo (*P. acutifolius*). This potentially reduces the inhibitory effect of CDK on retinoblastoma-related (RBR) protein by inhibiting CDK and therefore RBR inhibits E2Fa and E2Fb transcription factors which results in a down-regulation of their downstream genes observed in our expression study. The only detected gene from this pathway in Amadeus-77 (*P. vulgaris*) was a homolog of origin recognition complex 3 (ORC3) which was up-regulated indicating a positive effect on initiation of DNA replication at S-phase. Genes denoted with a star and in a blue box are found in this study. The blue and red arrows, indicate up- and down-regulated genes in this study, respectively. Source data underlying Supplementary Figure 9 are provided in the Dryad Digital Repository associated with this publication (https://doi.org/10.5061/dryad.6q573n5w2).

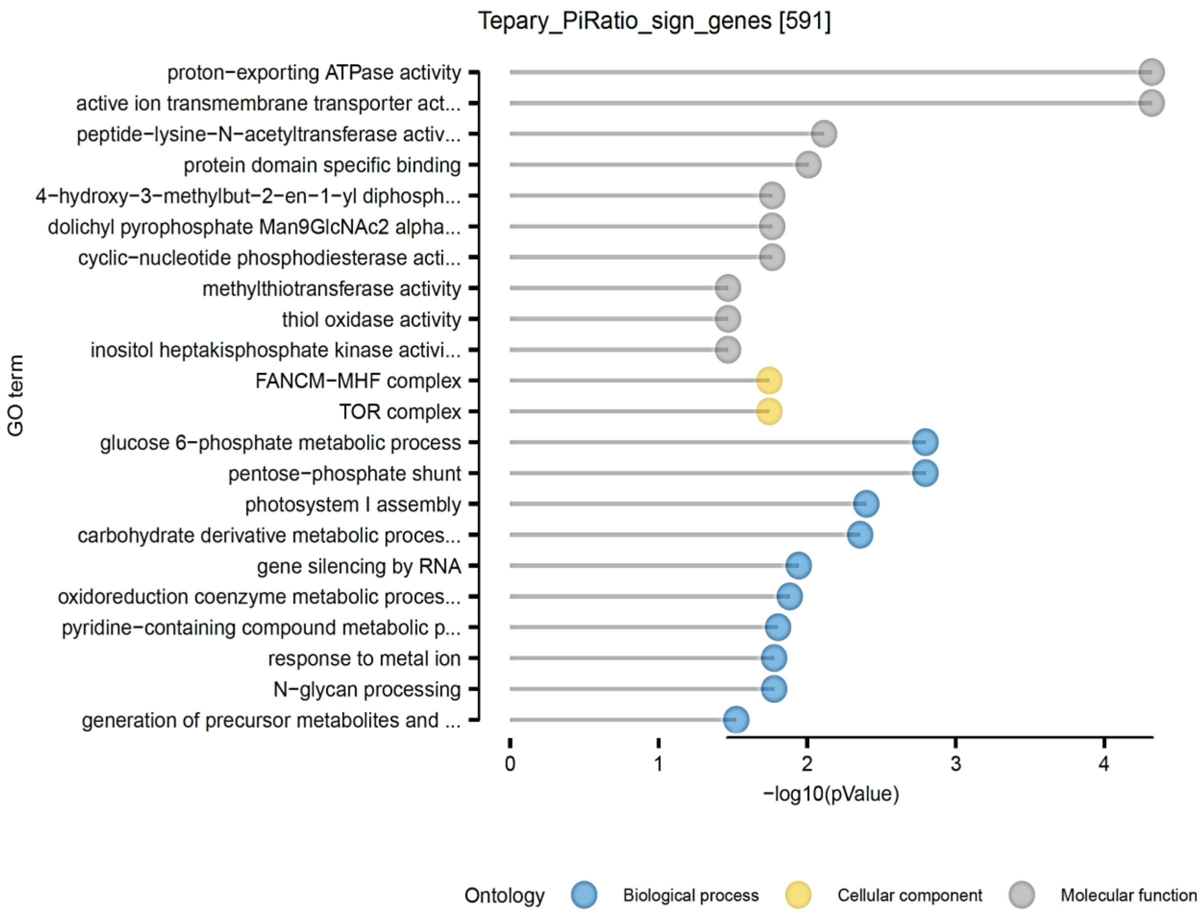

Supplementary Figure 10. Gene ontology term analysis of genes associated with low diversity in cultivated vs wild tepary bean; ion transportation is one of the major driving forces in domestication. The one-sided classic Fisher's exact test was used to assign a *P*-value to the GO term class. GO terms with *P*-value less than 0.05 were considered significant. Source data are provided as a Source data file.

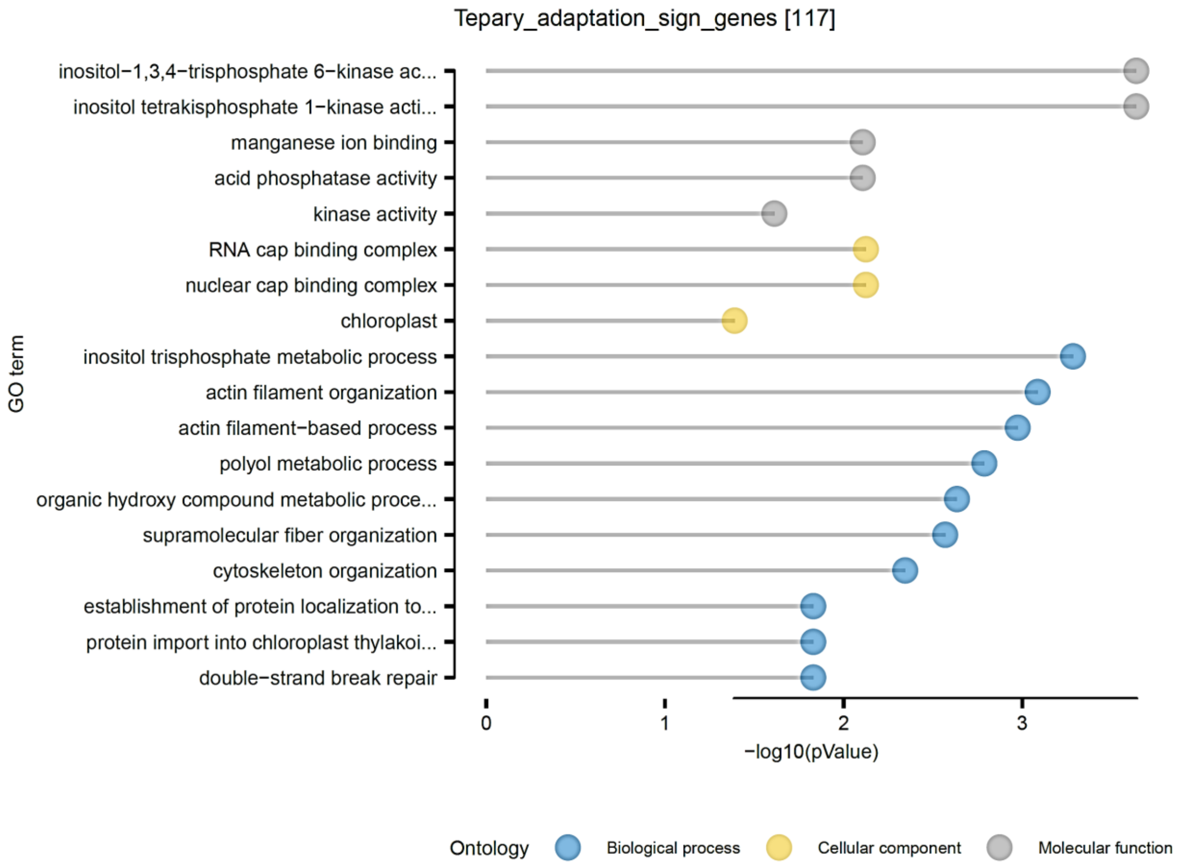

Supplementary Figure 11. Gene ontology term enrichment analysis of genes associated with fixed alleles in cultivated vs wild tepary bean; inositol phosphatase is a major driving force in adaptation. The one-sided classic Fisher's exact tests was used to assign a *P*-value to the GO term class. GO terms with *P*-value less than 0.05 were considered significant. Source data are provided as a Source data file.

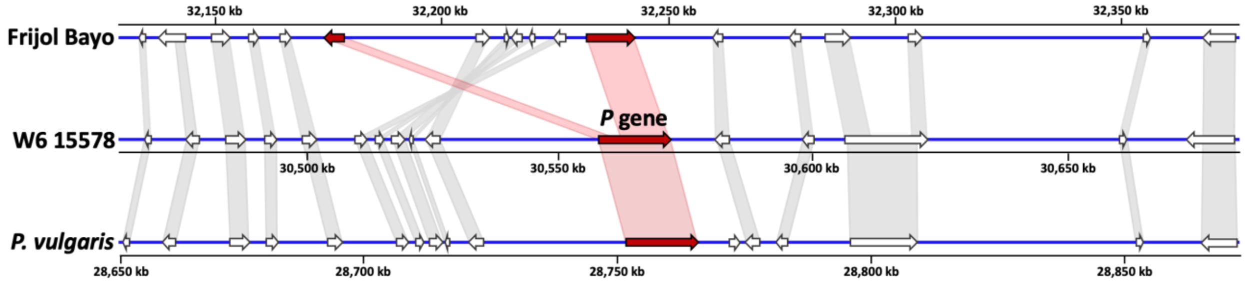

Supplementary Figure 12. Synteny between the *P* locus in common bean and tepary bean. A localized inversion at the *P* locus in Frijol Bayo is likely responsible for white seed-coat color, a domestication trait. The inversion spans a ~56 kb region on Pa07 and involves six genes. In Frijol Bayo, the wild type *P* gene (*Phacu.WLD.007G209100.1*) was disrupted by an inversion between exons 3 and 5, which reversed the orientation of exons 1-3. Exon 4 could not be identified in Frijol Bayo.

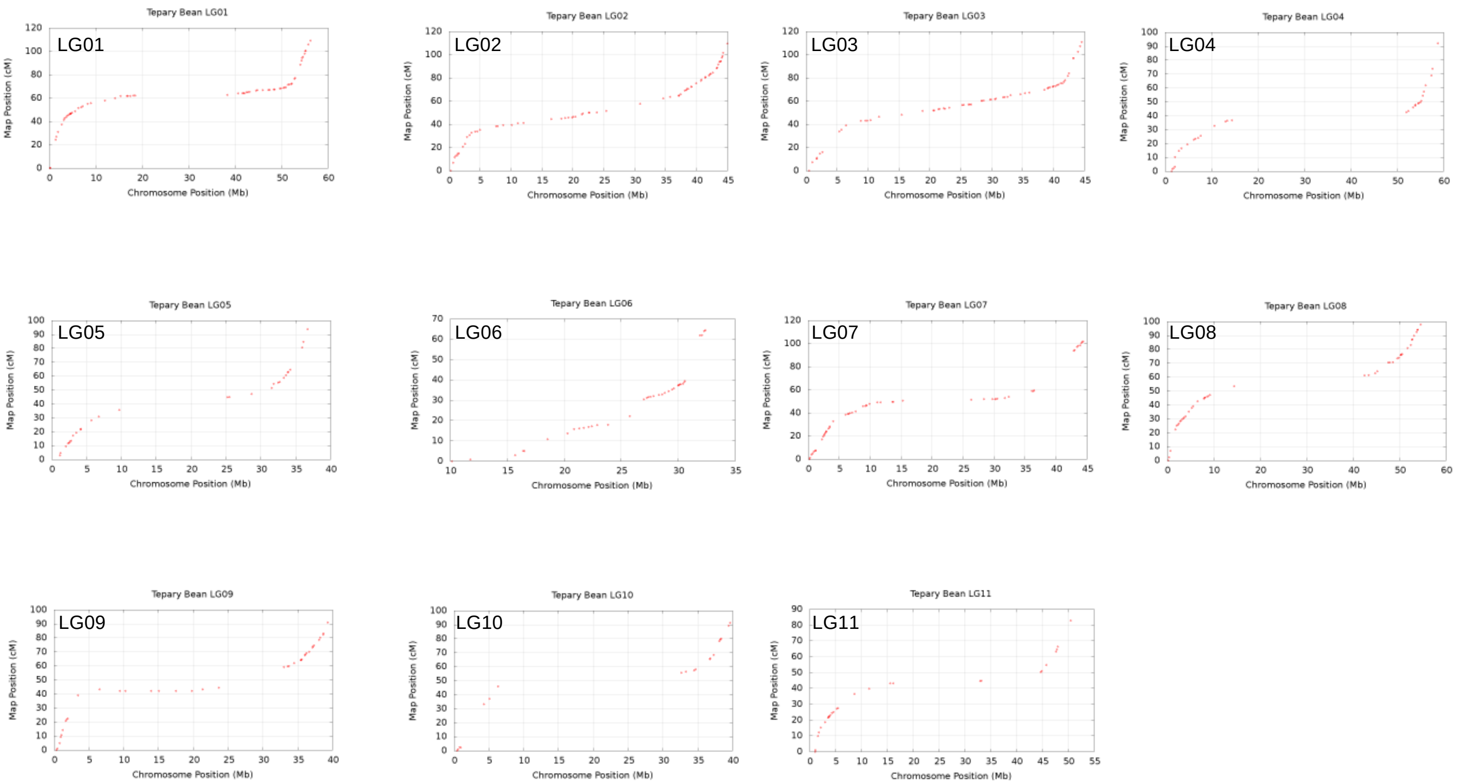

Supplementary Figure 13. Marker map placements on the cultivated *Phaseolus acutifolius* Frijol Bayo chromosomes 1 to 11.

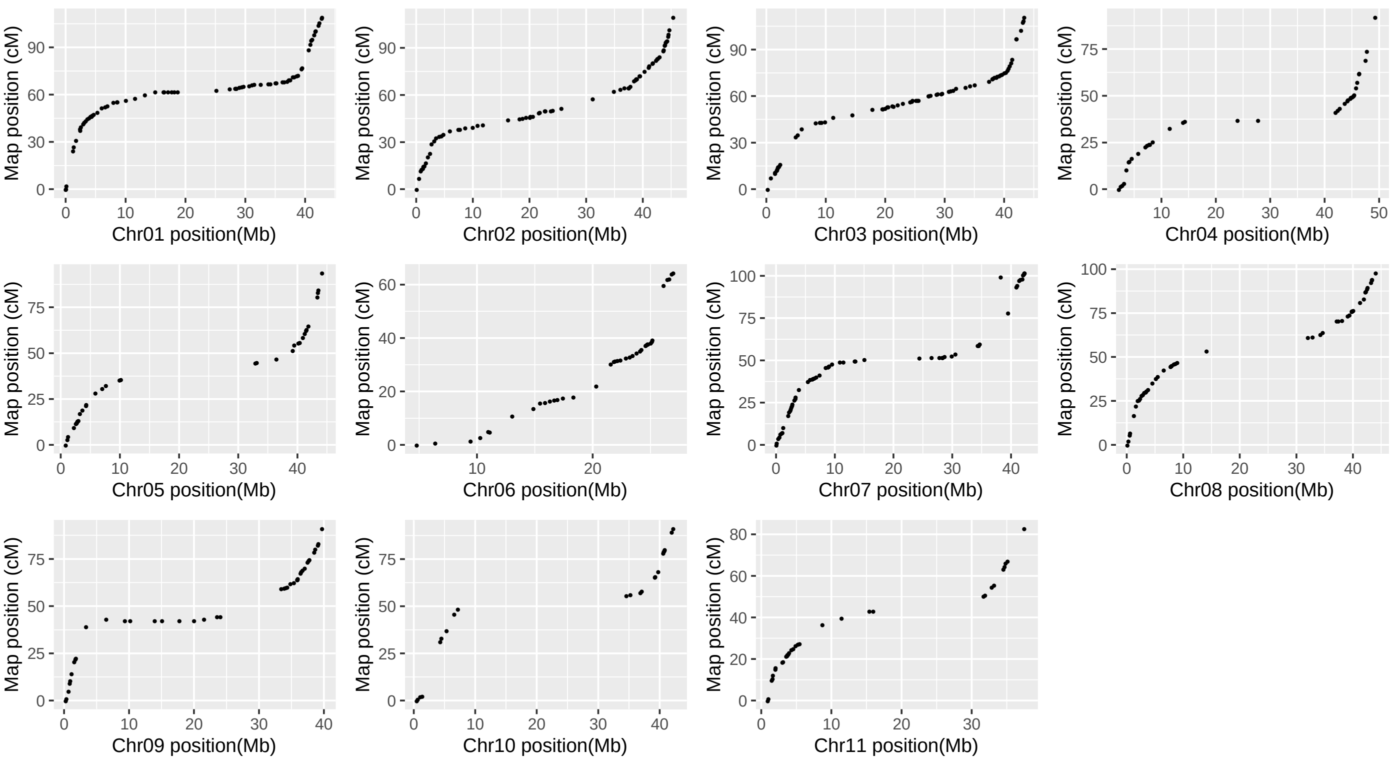

Supplementary Figure 14. Marker map placements on the wild *Phaseolus acutifolius* W6 15578 chromosomes 1 to 11. Based on the BR-06 genetic map of Gujaria-Verma *et al.* [1].

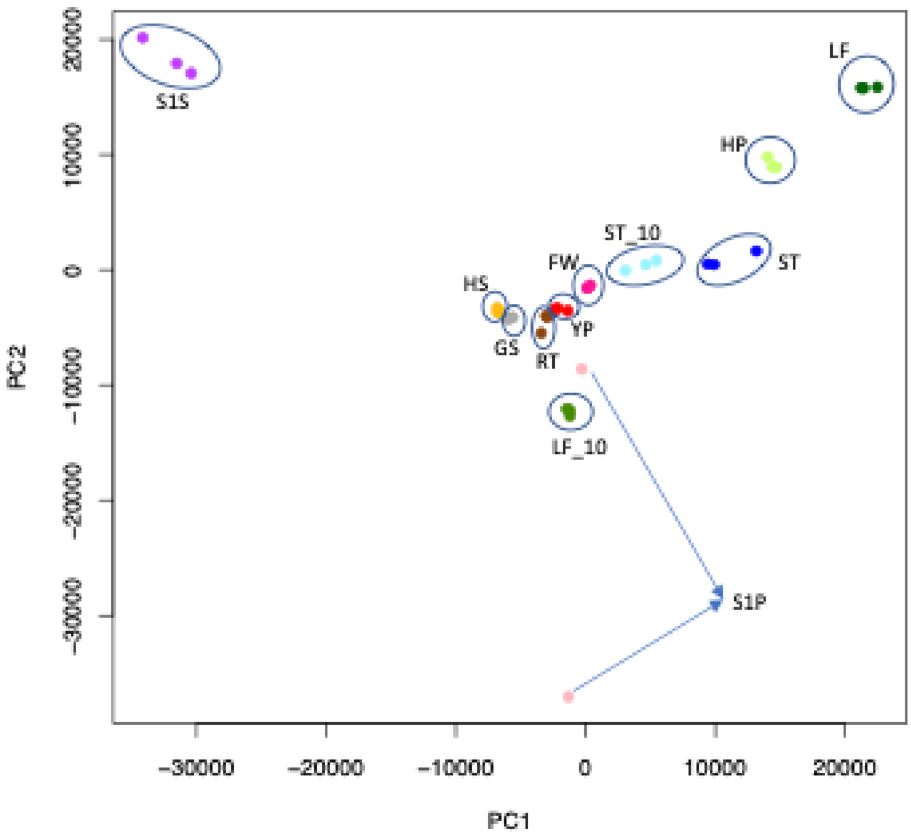

Abbreviation	Tissue	Color
FW	Open_flower_30day	deeppink
GS	Germinating seed	darkgrey
HP	Pod_shell_of_seed_3_4mm	chartreuse4
HS	Seed_3_4mm	darkgoldenrod1
LF	Leaf_30day	darkgreen
LF_10	Primary_leaf_10day	darkolivegreen1
RT	Root_10day	chocolate4
S1P	Pod_shell_of_seed_6_7mm	lightpink
S1S	Seed_6_7mm	darkorchid1
ST	Stem_30day	blue
ST_10	Stem_10day	cadetblue1
YP	Yound_pod_1_3_cM	red

Supplementary Figure 15. Principal Component Analysis (PCA) of transcriptomic data for 12 *P. acutifolius* developmental tissues. PCA was performed using Fragment Per Kilobase of transcript per Million mapped reads (FPKM). The first two principal components cumulatively account for 51.76 % of variation. Source data are provided as a Source data file.

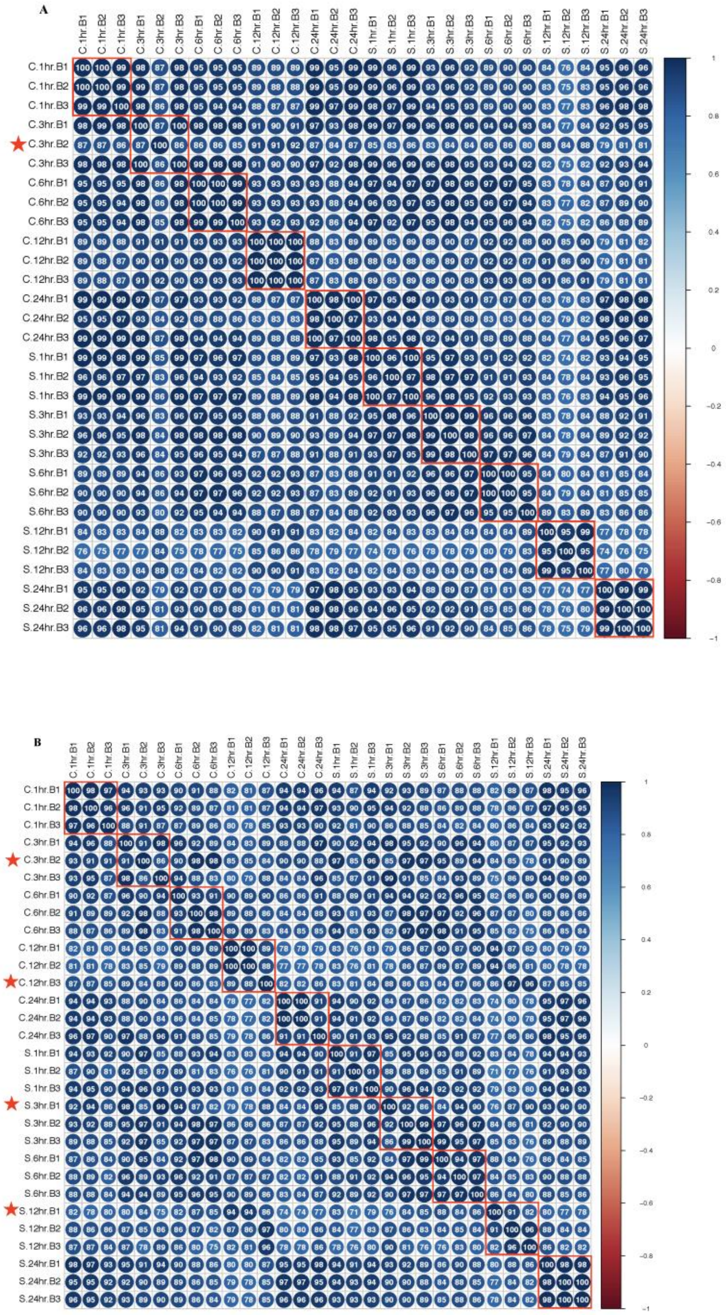

Supplementary Figure 16. Biological Replicate Pearson's Correlation Coefficient among the samples of time-course heat experiment for each genotype. All the biological replicates for each time point are placed inside a red rectangle. Samples with a correlation coefficient of less than 90% were removed. These samples are indicated with a red star next to them. A) Frijol Bayo (tepary bean samples) B) Amadeus-77 (common bean samples). C =control (29 °C day/ 20 °C night) / S=stress (32 °C day/ 27 °C night). Source data are provided as a Source data file.

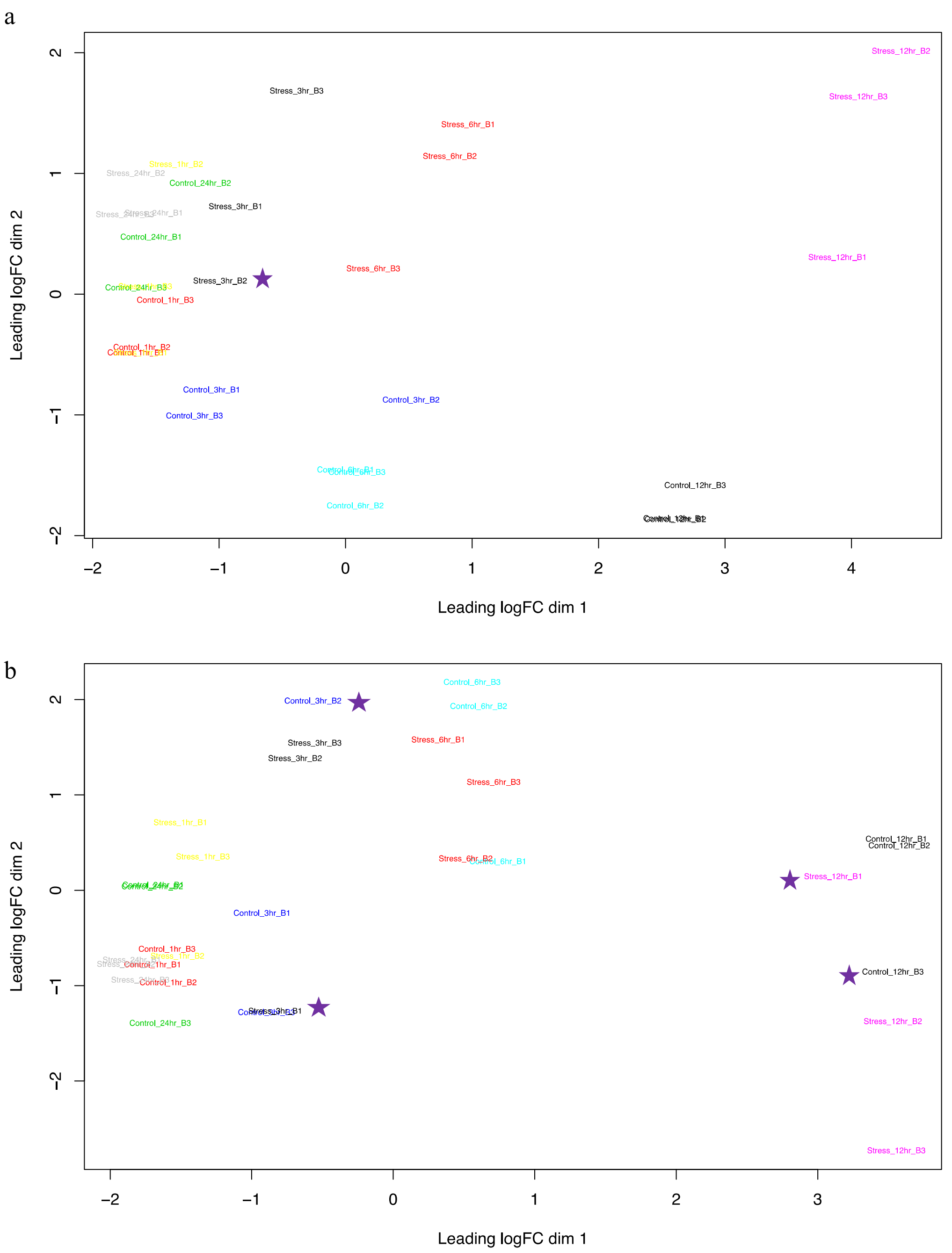

Supplementary Figure 17. Multidimensional Scaling (MDS) plot of transcriptome data under control (29 °C day/ 20 °C night) and moderate heat stress (32 °C day/ 27 °C night) conditions for a) tepary bean (Frijol Bayo) and b) common bean (Amadeus-77'). Samples with a star were removed for further analysis because they showed a Pearson correlation coefficient of less than 90% with the rest of the biological replicates in that treatment/time point. Source data are provided as a Source data file.

Supplementary References

1. Gujaria-Verma, N. *et al.* Gene-based SNP discovery in tepary bean (*Phaseolus acutifolius*) and common bean (*P. vulgaris*) for diversity analysis and comparative mapping. *BMC Genomics* 17, 239 (2016).

2. Gutierrez, C. The Arabidopsis cell division cycle. *Arabidopsis Book* 7, e0120 (2009).

3. Schmutz, J. *et al.* A reference genome for common bean and genome-wide analysis of dual domestications. *Nat. Genet.* 46, 707–713 (2014).
