# Supplementary material for: The tepary bean genome provides insight into evolution and domestication under heat stress
Source: Nat Commun. 2021 May 11;12:2638. doi: 10.1038/s41467-021-22858-x (PMC8113540; doi:10.1038/s41467-021-22858-x)
Supplement: Supplementary file 2 — Reporting Summary [file 41467_2021_22858_MOESM2_ESM.pdf]

nature research

Corresponding author(s): Porch, Bett, Buell, McClean

Last updated by author(s): 2020-20-12

Reporting Summary

Nature Research wishes to improve the reproducibility of the work that we publish. This form provides structure for consistency and transparency in reporting. For further information on Nature Research policies, see our Editorial Policies and the Editorial Policy Checklist.

Statistics

For all statistical analyses, confirm that the following items are present in the figure legend, table legend, main text, or Methods section.

n/a	Confirmed	
☐	☒	The exact sample size (*n*) for each experimental group/condition, given as a discrete number and unit of measurement
☐	☒	A statement on whether measurements were taken from distinct samples or whether the same sample was measured repeatedly
☐	☒	The statistical test(s) used AND whether they are one- or two-sided<br>*Only common tests should be described solely by name; describe more complex techniques in the Methods section.*
☒	☐	A description of all covariates tested
☐	☒	A description of any assumptions or corrections, such as tests of normality and adjustment for multiple comparisons
☐	☒	A full description of the statistical parameters including central tendency (e.g. means) or other basic estimates (e.g. regression coefficient) AND variation (e.g. standard deviation) or associated estimates of uncertainty (e.g. confidence intervals)
☐	☒	For null hypothesis testing, the test statistic (e.g. *F*, *t*, *r*) with confidence intervals, effect sizes, degrees of freedom and *P* value noted<br>*Give P values as exact values whenever suitable.*
☒	☐	For Bayesian analysis, information on the choice of priors and Markov chain Monte Carlo settings
☒	☐	For hierarchical and complex designs, identification of the appropriate level for tests and full reporting of outcomes
☐	☒	Estimates of effect sizes (e.g. Cohen's *d*, Pearson's *r*), indicating how they were calculated

Our web collection on statistics for biologists contains articles on many of the points above.

Software and code

Policy information about availability of computer code

Data collection

No software used in data collection

Data analysis

ARACNeAP (version 20190423): Gene expression network analysis software.
ARROW (version 2.2.2): Error correction for genome reads.
Augustus (v3.2.2): Gene modeling software.
BUSCO v3.0.2b: Benchmarking single copy orthologs to assess gene representation in a genome.
BWA (V0.7.1; 0.7.15) Burrows-Wheeler Alignment Tool.
CAFE (v4.2.1: Gene family evolutionary analyses.
COILS (ver. 2.2): Software to predict coil domains in proteins.
cor function: PRogram in R to calculate Pearson's correlations
Cutadapt (v 1.16; v1.18): Removes adaptors and low quality sequences from raw sequence reads.
Cufflinks (v 2.2.1; v2.8): Expression abundance estimations
Cytoscape (v3.7.1): Network visualization software
DenovoMagic -V2 NRGene’s whole-genome assembly software for Illumina short-read and 10X Chromium data.
DESeq2 (v1.22.2): Differential gene expression analyses
FastQC (v 0.11.5: Sequence read quality assessment software.
findGSE (v0.1.0): K-mer analysis package.
GATK’s UnifiedGenotyper v3.6-0-g89b7209: Software to identify genotypes.
HISAT2 (v2.1.0): Aligns RNA-sequencing reads to a genome.
Jellyfish v2.2.6: K-mer analyses for whole genome shotgun sequencing data.
JUICER version 1.5.6: Chromatin contact software with HiC reads.
HMMER (v3.1b2; v3.2.1): Searches databases for similar sequences.

HTseq (v0.9.1; v0.11.2): Program to count aligned reads
InterProScan (v 5.34-73.0): Domain and motif finding software
Kallisto (v0.45.0): Transcript abundance software.
Limma: Package in R for differential gene expression
Maker-P v2.31.10. Gene annotation pipeline tailored for plant genomes.
Mclust version 5.4.6: Mclust is an R library for clustering, classification, and density estimation based on Gaussian mixture modelling
MCScanX V2 Multiple Collinearity Scan toolkit.
MECAT version 1.0: Genome assembly software
MEGA (v7.0): Phylogenetic analysis package
MUSCLE (ver 3.4): Multiple sequence alignment software
OrthoFinder2 (v2.2.7): Orthologous/paralogous cluster identification.
pairwise_kaks.PLS (v1.8): Software to estimate synonymous substitutions
PASA2 (v2.2.0 and v2.3.3): Gene modeling software.
prcomp: Function in R to calculate principal components
ProtExcluder v1.1: Identifies non-transposable element related sequences in a list of repetitive sequences.
psych: Program in R to calculate P-values
R (v3.51): Project for statistical computing.
RepeatMasker v4.0.6: Masks genomes for repeat sequences.
RepeatModeler v1.0.8: Identifies repetitive sequences in DNA sequence.
seqtk (v 1.0.r82): Sequence processing software
snpEff (v4.0): Program to determine SNP impact on protein function
STAR V2.7.1a Spliced transcripts alignment software.
STRUCTURE (v2.3.4): Program to identify population structure.
TopGO (v2.36.0; v2.38.1): Bioconductor package to test for enrichment of gene ontologies
Trimmomatic V0.38 Read trimming tool for Illumina NGS data.
Trinity (v2.6.6 and v2.8.4): Generates transcript assemblies from RNA-sequencing reads.
UpsetR (v1.4.0): Package within R to plot orthologous groups

For manuscripts utilizing custom algorithms or software that are central to the research but not yet described in published literature, software must be made available to editors and reviewers. We strongly encourage code deposition in a community repository (e.g. GitHub). See the Nature Research guidelines for submitting code & software for further information.

Data

Policy information about availability of data

All manuscripts must include a data availability statement. This statement should provide the following information, where applicable:

- Accession codes, unique identifiers, or web links for publicly available datasets
- A list of figures that have associated raw data
- A description of any restrictions on data availability

Data supporting the findings of this work are available within the paper and its Supplementary Information files. Raw sequence reads are available in the National Center for Biotechnology Information Sequence Read Archive under BioProject ID “PRJNA607288” (https://www.ncbi.nlm.nih.gov/sra/?term=PRJNA607288). Large associated datasets include (i) genome assembly, gene annotation (genes, gene models, transcripts, peptides), and associated gff files; (ii) gene expression matrices and differentially expressed genes; and (iii) orthologous groups are available in the Dryad Digital Repository (https://doi.org/10.5061/dryad.6q573n5w2). The genome assemblies with their annotation are available at Phytozome (https://phytozome-next.jgi.doe.gov/info/Pacutifolius_v1_0; https://phytozome-next.jgi.doe.gov/info/PacutifoliusWLD_v2_0). Data supporting the findings of this work are available within the paper and its Supplementary Information files. The source data underlying Figures as well as Supplementary Figures are provided in the Source Data file and in the large files deposited in the Dryad Digital Repository. External datasets used in this study include: G. max proteome (EnsemblPlants; Glycine_max_v2.1.pep.all.fa; https://plants.ensembl.org/); PFAM (v32; http://pfam.xfam.org/); Phytozome genome/proteomes (A. thaliana proteome (TAIR10); Cajanus cajan (v1); Glycine max (Wm82.a2.v1); Medicago truncatula (Mt4.0v1); P. vulgaris V2.1; Vigna unguiculata (v1.1); https://phytozome.jgi.doe.gov/pz/portal.html); RepBase (v20150807) Viridiplantae repeats; Swiss-Prot (2015_08; https://www.uniprot.org/); Plant Transcription Factor Database v.4.0 (planttfdb.cbi.pku.edu.cn/prediction.php). A reporting summary for this Article is available as a Supplementary Information file. The datasets analyzed during the current study are available from the corresponding author upon request. The germplasm is available from the U.S. Department of Agriculture Germplasm Resources Information Network (https://www.ars-grin.gov/).

Field-specific reporting

Please select the one below that is the best fit for your research. If you are not sure, read the appropriate sections before making your selection.

☒ Life sciences ☐ Behavioural & social sciences ☐ Ecological, evolutionary & environmental sciences

For a reference copy of the document with all sections, see nature.com/documents/nr-reporting-summary-flat.pdf

Life sciences study design

All studies must disclose on these points even when the disclosure is negative.

Sample size	For generation of genome assemblies, only a single accession can be used. We generated the genome assembly of one cultivated and one wild accession of tepary bean.
Data exclusions	One sample from the tepary RNA-seq libraries (Control_3h_B2) and four samples from the common bean RNA-seq libraries (Control_3h_B2, Control_12h_B3, Stress_3h_B1, Stress_12h_B1) were excluded based on Pearson's Correlation Coefficients of less than 0.9.
Replication	RNA-sequencing was performed in triplicate and was examined for reproducibility using Pearson's Correlation Coefficient analyses.
Randomization	Samples for gene expression profiling were randomized in the growth chamber.
Blinding	Blinding was not applicable in this study as it did not involve sampling of populations.

Reporting for specific materials, systems and methods

We require information from authors about some types of materials, experimental systems and methods used in many studies. Here, indicate whether each material, system or method listed is relevant to your study. If you are not sure if a list item applies to your research, read the appropriate section before selecting a response.

Materials & experimental systems

n/a	Involved in the study	
☒	☐	Antibodies
☒	☐	Eukaryotic cell lines
☒	☐	Palaeontology and archaeology
☒	☐	Animals and other organisms
☒	☐	Human research participants
☒	☐	Clinical data
☒	☐	Dual use research of concern

Methods

n/a	Involved in the study	
☒	☐	ChIP-seq
☒	☐	Flow cytometry
☒	☐	MRI-based neuroimaging
