# Supplementary material for: The tepary bean genome provides insight into evolution and domestication under heat stress
Source: Nat Commun. 2021 May 11;12:2638. doi: 10.1038/s41467-021-22858-x (PMC8113540; doi:10.1038/s41467-021-22858-x)
Supplement: Supplementary file 3 — Description of Additional Supplementary Files [file 41467_2021_22858_MOESM3_ESM.pdf]

Description of Additional Supplementary Files

Supplementary Data 1. BUSCO analysis of genome quality and completeness for A) cultivated (Frijol Bayo) and B) wild tepary (W 615578) genotypes.

Supplementary Data 2. Repetitive sequence content in *Phaseolus vulgaris* and two *Phaseolus acutifolius* genomes.

Supplementary Data 3. Divergence time estimated based on Ks (synonymous substitutions) distributions.

Supplementary Data 4. Differential gene expression of heat shock response genes in *P. vulgaris* and *P. acutifolius* following prolonged heat stress of 36 C day/32 C.

Supplementary Data 5. Differential gene expression for leaf tissues under prolonged and elevated heat stress conditions of 36 C day and 32 C night in *P. vulgaris* and *P. acutifolius*.

Supplementary Data 6. Top 50 gene ontology terms from GO enrichment analysis of differentially expression genes in Amadeus-77 (*P. vulgaris*) for each up- and down-regulated clusters at each time point.

Supplementary Data 7. Top 50 gene ontology terms from GO enrichment analysis of differentially expression genes in Frijol Bayo (*P. acutifolius*) for each up- and down-regulated clusters at each time point.

Supplementary Data 8. *P. acutifolius* (Frijol Bayo) NB-ARC domain containing genes

Supplementary Data 9. *P. vulgaris* NB-ARC domain containing genes.

Supplementary Data 10. *P. acutifolius* (Frijol Bayo) transcription factors.

Supplementary Data 11. *P. vulgaris* transcription factors.

Supplementary Data 12. Distribution of transcription factors across families for *P. acutifolius* landrace (Frijol Bayo) and wild (W6 15578), and *P. vulgaris*.

Supplementary Data 13. Modular structure of NB-ARC protein family members for *P. acutifolius* cultivated (Frijol Bayo) and wild (W6 15578), and *P. vulgaris* landrace.

Supplementary Data 14.Integrated domains located in *P. acutifolius* landrace (Frijol Bayo), *P. acutifolius* wild (W6 15578), and *P. vulgaris* landrace gene models.

Supplementary Data 15. Distribution of NB-ARC proteins in *Phaseolus acutifolius* (Frijol Bayo) and *Phaseolus vulgaris* clusters.

Supplementary Data 16. Recipricol best hits between *P. acutifoloius* (Frijol Bayo) and *P. vulgaris* NB-ARC domain containing proteins.

Supplementary Data 17. 55 genotypes which best represented the diversity among the 8 subpopulations of the tepary diversity panel and selected for resequencing.

Supplementary Data 18. Regions with reduction or complete loss in diversity during tepary domestication.

Supplementary Data 19. Frijol Bayo developmental tissues used for expression support of genome annotation process.
